# Lipoxins as Modulators of Diseases

**DOI:** 10.3390/cells14161244

**Published:** 2025-08-12

**Authors:** Uzma Saqib, Monika Pandey, Anjali Vyas, Preeti Patidar, Sumati Hajela, Asgar Ali, Meenakshi Tiwari, Sutripta Sarkar, Neelam Yadav, Shivani Patel, Deepali Shukla, Grace N. Lienemann, Fletcher A. White, Herney Andrés García-Perdomo, Mirza Saqib Baig, Ganesh V. Halade, Krishnan Hajela, Sadhana Sharma, Alexander G. Obukhov

**Affiliations:** 1 School of Life Sciences, Devi Ahilya Vishwavidyalaya, Khandwa Road Campus, Indore 452001, Madhya Pradesh, India; uzmas2024@gmail.com (U.S.); mppandeymonika32@gmail.com (M.P.); anjali14vyas12@gmail.com (A.V.); neelamy24@gmail.com (N.Y.); itspatelshivani@gmail.com (S.P.); 2 Dr. Vikram Sarabhai Institute of Cell and Molecular Biology, The Maharaja Sayajirao University of Baroda, Vadodara 390002, Gujarat, India; preeti.patidar1988@gmail.com; 3 Department of Biotechnology, Mahakaushal University, Jabalpur 482003, India; sumati.hajela@gmail.com; 4 Department of Biochemistry, All India Institute of Medical Sciences, Patna 801507, Bihar, India; drasgarasli@aiimspatna.org; 5 Department of Center for Advance Research, King George’s Medical University, Lucknow 226003, Uttar Pradesh, India; 6 Post Graduate Department of Food and Nutrition, Barrackpore Rastraguru Surendranath College, Kolkata 700120, West Bengal, India; sarkar.sutripta@gmail.com; 7 Department of Microbiology, College of Life Sciences, Cancer Hospital and Research Institute, Gwalior 474009, Madhya Pradesh, India; trustdeepali3@gmail.com; 8 Department of Anatomy, Cell Biology & Physiology, Indiana University School of Medicine, Indianapolis, IN 46202, USA; 9 Department of Anesthesia, Indiana University School of Medicine, Indianapolis, IN 46202, USA; fawhite@iu.edu; 10 Stark Neurosciences Research Institute, Indiana University School of Medicine, Indianapolis, IN 46202, USA; 11 UROGIV Research Group, Department of Surgery, School of Medicine, Universidad del Valle, Cali 72824, Colombia; herney.garcia@correounivalle.edu.co; 12 Division of Urology/Urooncology, Department of Surgery, School of Medicine, Universidad del Valle, Cali 72824, Colombia; 13 Mehta Family School of Biosciences and Biomedical Engineering, Indian Institute of Technology Indore (IITI), Indore 453552, Madhya Pradesh, India; msb.iit@iiti.ac.in; 14 Heart Institute, Division of Cardiovascular Sciences, Department of Internal Medicine, University of South Florida, Tampa, FL 33602, USA; ghalade@usf.edu

**Keywords:** lipoxins, eicosanoids, inflammation, therapeutic agents, host defense, synthetic lipoxins, lipoxygenases, immunomodulation

## Abstract

Lipoxins were discovered 40 years ago, and since then, their beneficial roles for human health have been confirmed in numerous studies. These small molecules belong to the eicosanoid class of compounds, which are generated metabolically by lipoxygenases. Lipoxins are released during various diseases and conditions, including but not limited to systemic inflammation, infection, asthma, cancer, diabetes, and cardiovascular disorders. Recently, several synthetic lipoxin analogs have been developed that also exhibit potent anti-inflammatory properties. In this review, we discuss the inflammation-resolving roles of lipoxins in various major diseases. Further, we summarize the latest reports on the use of synthetic lipoxins as potential therapeutic agents and discuss the role of aspirin-dependent lipoxin production in alleviating various diseases, including cancer.

## 1. Introduction

The term “lipoxin” was coined by Drs. Serhan, Hamberg, and Samuelsson in 1984. They discovered that human leukocytes could produce 5,6,15L-trihydroxy-7,9,11,13-icosatetraenoic acid (lipoxin A or LAX4) and 5D,14,15L-trihydroxy-6,8,10,12-icosatetraenoic acid (lipoxin B or LAXB) via lipoxygenase (LOX)-dependent conversion of arachidonic acid [1,2]. Later, two additional members of the lipoxin family were identified, which were synthesized by cells in the presence of aspirin. They had different trihydroxyeicosatetraenoic acid moieties and were named 15-epi-LXA4 ((5S,6R,7E,9E,11Z,13E,15R)-5,6,15-trihydroxyicosa-7,9,11,13-tetraenoic acid) and 15-epi-LXB4 ((5S,6E,8Z,10E,12E,14R,15R)-5,14,15-trihydroxyicosa-6,8,10,12-tetraenoic acid) [3,4,5].

LXs are produced by various cell types in the human body during host responses to inflammation, infection, or injury. They exhibit anti-inflammatory and immunoregulatory properties and can modulate both innate and adaptive immune responses. Primarily, LXs contribute to resolving acute and chronic inflammation by facilitating neutrophil clearance from the site of infection and reducing pro-inflammatory mediator release from macrophages [6]. LXs have also been reported to have anti-apoptotic effects in macrophages via the activation of the PI3K/Akt and ERK/Nrf-2 pathways [7], increasing macrophage viability. Conversely, LXs are known to stimulate non-phlogistic phagocytosis of apoptotic neutrophils by macrophages [8]. Furthermore, LXs may attenuate memory B cell responses by activating the G-protein coupled ALX/FPR2 receptor that binds LXA4 with a high affinity [9,10]. All these LX effects contribute to the resolution of inflammation and restoration of tissue homeostasis [11].

LXs are the members of the superfamily of specialized pro-resolving mediators (SPMs), which also include resolvins, protectins, and maresins [12]. In contrast to LXs, which are derived from arachidonic acid, resolvins, protectins, and maresins are produced via lipoxygenase-mediated conversion of omega-3-fatty acids, such as docosahexaenoic acid (DHA). This results in resolvins, protectins, and maresins containing six double bonds, two more than LXs. The biosynthesis pathways and physiological properties of resolvins, protectins, and maresins are described in detail in a recent review by Ferreira et al. [13]. The current review focuses only on LXs and summarizes the literature on their role in resolving inflammation and improving outcomes in various diseases and discusses the potential clinical applications of exogenous LXs in alleviating the severity of diseases.

## 2. Biosynthesis of Lipoxins

LXs are derived from arachidonic acid (AA). The two main members of the LX family are LXA4 (6R,15S-trihydroxy-7,9,13-trans-11-cis-eicosatetraenoic acid) and its positional isomer LXB4 (5S,14R,15S-trihydroxy-6,10,12-trans-8-cis-eicosatetraenoic acid). Although multiple cell types can produce and secrete LXs, transcellular metabolism is the primary pathway for LX formation in human tissues when special cell types produce and release LX intermediates, which are then taken up by neighboring cells to complete LX biosynthesis. There are two primary transcellular pathways for LX production. The first pathway involves the bidirectional interplay of platelets and neutrophils through transcellular metabolism. In this case, LXA4 and LXB4 are produced either when neutrophils convert arachidonates derived from the FMLP/thrombin-stimulated platelets or when platelets convert Leukotriene A4 (LTA4) derived from neutrophils [14]. The second pathway of LX production involves the interplay between neutrophils and epithelial cells. This process involves the 15- and 5-lipoxygenase (LOX) enzymes. An alternative route involves the 15-LOX-mediated conversion of leukotrienes (LTs) into 5S, 6S, and 15S-epoxytetraenes, which are then transformed into LXs [15]. Additionally, a distinct aspirin-triggered biosynthetic pathway leads to different types of LXs, which are referred to as 15R epimers, or 15-epi-LXs (aspirin-triggered lipoxins, ATLs) and are produced via endothelial cell–leukocyte interactions [16]. During aspirin-triggered LX production, aspirin-acetylated cyclooxygenase-2 (COX-2) metabolizes arachidonates in endothelial cells into 15(R)-hydroxyeicosatetraenoic acid, which is then converted into unnatural 15-epi-LXs by lipoxygenases in leukocytes [3,17]. 15-epi-LXs are more resistant to prostaglandin dehydrogenase metabolism and, therefore, are more stable compared to LXs [18].

5-LOX activity, which is primarily present in peripheral neutrophils, monocytes, and monocyte-derived macrophages, is important for the production of not only LXs but also LTs [17]. LXs and LTs are both eicosanoids derived from arachidonic acid, but they differ in their molecular structures and biological functions. LTs are known for their pro-inflammatory effects, such as promoting leukocyte recruitment, vascular permeability, and bronchoconstriction (for review, see [19]), whereas LXs have anti-inflammatory activity [20]. This suggests that there may be an intricate interplay between these two groups of bioactive molecules and that the delicate balance between them may determine physiological outcomes. Notably, in platelets, LXs can be produced from leukotriene A4 (LTA4) derived from neutrophils in a 12-LOX-dependent manner [21]. Thus, besides 5-LOX, either 12-LOX, 15-LOX, or aspirin-acetylated COX2 is needed for LX biosynthesis, and it involves transcellular eicosanoid transfer.

Readers interested in a more detailed description of the biochemical pathways involved in LX production are referred to a review by Drs. Folco and Murphy [17] that provides an excellent overview of LX and leukotriene biosynthesis pathways.

## 3. LX Receptors

LXs and ATLs exhibit their physiological effects after binding to the G protein-coupled lipoxin A4 receptor (ALX)/Formyl peptide receptor 2 (FPR2), or the ALX/FPR2 receptor, which is described in depth elsewhere [22]. The ALX/FPR2 receptor is a homodimer [23] and may be coupled either to the G_i/o_ or G_q/11_ heterotrimeric G-proteins [22]. This receptor can also form heterodimers with FPR1 and FPR3 and may operate in a ligand-biased fashion [23]. The ALX/FPR2 receptor is known for its complex biological functions and plays a crucial role in regulating the anti-inflammatory and pro-resolving properties of LXs, particularly lipoxin A4 (LXA4). Various ligands can bind to this receptor by interacting with different binding sites on the ALX/FPR2 receptor protein, resulting in ligand-biased signaling, with amyloid-β and Prion protein causing pro-inflammatory reactions, whereas SPMs causing anti-inflammatory reactions (for review, see [24]). In addition to ALX/FPR2, lipoxins can activate other receptors, such as GPR32, the cysteinyl-leukotriene receptor, and the cytoplasmic aryl hydrocarbon receptor, which in turn trigger different intracellular signaling pathways [25,26]. The pharmacological activation of these receptors may have promising anti-inflammatory effects on humans. For example, in the bronchi, LXs can competitively inhibit the interaction of pro-inflammatory cysteinyl-leukotrienes with their receptor, resolving inflammation [27].

LX receptors are widely expressed on epithelial cells, neurons, astrocytes, neural stem cells, and microglia [27,28,29]. Therefore, LXs may play a role in regulating inflammation throughout the body. For instance, in microglial cells, ALX/FPR2 receptor expression enables ATLs to inhibit the release of pro-inflammatory cytokines and other mediators in response to lipopolysaccharide (LPS), a bacterial cell wall component, that can cause inflammation [30]. This is achieved by modulating signaling events, such as NF-κB nuclear translocation and the activation of MAPK, which occur during inflammation [30]. The receptor is also expressed by neutrophils, eosinophils, monocytes, macrophages, T cells, and various types of epithelial cells and is involved in multiple physiological processes, pathophysiological conditions, and the resolution of inflammation [26].

LXA4 often teams up with annexin-derived peptides to trigger phagocytosis, and the receptor ALX/FPR2 must be expressed and then internalized during this process [31]. A crucial step in resolving inflammation is the stimulation of non-inflammatory phagocytosis of apoptotic cells by macrophages, which is facilitated by LXs such as LXA4. This phagocytic action requires receptor internalization and is reliant on protein kinase C (PKC) activation. The intricate trafficking and activity of receptors play a crucial part in the resolution of inflammation [31].

Glucocorticoids (GCs) exhibit potent anti-inflammatory properties, but they may also exhibit pro-inflammatory effects [32]. For example, GCs may stimulate 5-lipoxygenase, an enzyme necessary for the production of pro-inflammatory leukotrienes, such as LTB4. Hashimoto et al. used a model of LTB4-induced mouse dermatitis to examine the relationship between glucocorticoids and lipoxin A4 receptors and found that the anti-inflammatory effects of glucocorticoids may be attributable, in part, to the upregulation of ALX/FPR2, suggesting that the lipoxin system may serve as a negative regulator for LTB4 signaling [33]. Furthermore, the therapeutic potential of the glucocorticoid-regulated anti-inflammatory mediator annexin A1 (ANX-A1) has been recently recognized in various systemic inflammatory disorders. ANX-A1 binds to and activates the family of formyl peptide receptors (FPRs) to inhibit neutrophil activation, migration, and infiltration. Consistently, recent research demonstrated that ANX-A1 and its peptide mimetic (Ac2-26, CGEN-855A) exhibited the anti-inflammatory and cardioprotective effects, preserving myocardial viability following ischemia–reperfusion injury [34].

Despite the marked progress in the field, there have also been some conflicting reports on LX receptor function that can be attributed to several factors. Firstly, it may be owing to the variation in experimental models involving either different in vitro cell cultures, the use of various animal species, or human samples. Divergent outcomes may be owing to inherent differences in immune responses and receptor expression patterns [35]. For instance, Colby et al. demonstrated strain-specific differences in Cryptococcus neoformans clearance linked to variations in LXA4 biosynthesis and ALX/FPR2 receptor expression in mice, highlighting the importance of the host’s genetic background in shaping LX effects [36]. Secondly, the concentration and subtype of LXs administered (e.g., LXA4 vs. 15-epi-LXA4, or LXB4) may influence their receptor binding and downstream signaling. LXB4, for example, exhibited more potent effects on neutrophil function than LXA4 in atherosclerotic patients, suggesting differential receptor affinity and function among LX analogs [37]. Thirdly, receptor expression levels—particularly of ALX/FPR2—vary across tissues and pathological conditions. Receptor downregulation may impair LX signaling in certain diseases like asthma or advanced atherosclerosis, limiting their anti-inflammatory efficacy [38]. Fourthly, LXs, ATLs, or their synthetic mimetics may exert diverse cellular effects by engaging the same ALX/FPR2 receptor in a ligand-biased manner. In this case, different receptor agonists, due to a unique ligand–receptor interaction profile, preferentially activate a specific intracellular pathway, such as G-protein (Gi), pERK1/2, or β-arrestin signaling, leading to distinct outcomes in cells [39]. For example, ATL and synthetic LX analogs, BML-111, are known to exhibit ligand bias: ATL preferentially activates β-arrestin-mediated anti-inflammatory pathways, while BML-111 shows stronger inhibition of MAPK signaling [23,40,41]. Understanding this ligand bias is critical for designing selective therapies. It was proposed that by tailoring lipoxin-based treatments to the receptor context and signaling profile of a disease, one can maximize therapeutic benefits while minimizing off-target effects [40].

Additionally, the stage of disease may play a role. For example, early-phase sepsis may be exacerbated by LXs, dampening the necessary immune response. At the same time, late-phase administration improves outcomes by curbing excessive inflammation, as shown by Sordi et al. in pneumoseptic mice [42]. Finally, the pharmacokinetics and metabolic stability of native versus synthetic LXs and the co-administration of drugs like aspirin, which triggers 15-epi-LX production, can further modulate the observed therapeutic effects [43]. Acknowledging and dissecting these variables is essential for appreciating the nuanced role of LXs across varied disease contexts.

## 4. Synthetic LX Modulators

The ability of exogenous LXs to exhibit anti-inflammatory effects makes them an attractive option for treating various inflammatory conditions. However, the conversion of LXs to 15-oxo-LXs by prostaglandin dehydrogenase (PGDH) limits LX functional activity [44], although the naturally occurring aspirin-triggered counterparts 15-epi-LXA4 and 15-epi-LXB4 demonstrate increased resistance to degradation. Thus, modifying the original LX structure to enhance LX stability, while preserving its anti-inflammatory properties, may be useful for improving the molecule’s therapeutic potential [45]. Over the last 30 years, researchers have synthesized several synthetic LX analogs. Thus far, four generations of lipoxin analogs [46] have been developed and tested in preclinical models.

The LXA4 molecule is divided into three regions: a lower carbon chain, an upper carbon chain, and a triene core [47]. The first generation of lipoxin analogs was designed using naturally occurring ATLs with modifications in the lower chain, including methyl and phenoxyl groups. These functional groups protected the molecules against 15-hydroxyprostaglandin dehydrogenase (15-PGDH)-mediated degradation [48]. These analogs are structural mimics of endogenous lipoxins, such as LXA4 and LXB4, with minimal chemical modifications. They maintain basic biological activity and are generally well tolerated. However, their effectiveness is limited due to rapid metabolic degradation, particularly by the enzyme 15-PGDH, as well as their low receptor selectivity. Consequently, they are primarily used in early-phase research or mechanistic studies [48]. In vitro, the molecules showed anti-inflammatory and pro-resolving bioaction, similar to native LX, including the inhibition of neutrophil recruitment, modulation of T cell responses, and enhanced FPR2-dependent macrophage efferocytosis. They also altered vascular permeability and inhibited neutrophil trafficking in acute inflammatory models [49,50].

ATLa (15-epi-16-(para-fluorophenoxy)-LXA4-methyl ester), an aspirin-triggered lipoxin analog, has been reported to protect against acute kidney damage by modulating cytokine and chemokine signaling networks [51]. Despite their good bioavailability and resistance to PGDH-mediated metabolism, the compounds were susceptible to β-oxidation, resulting in short plasma retention times for this type of LX synthetic analogs [52].

**Figure 1:**
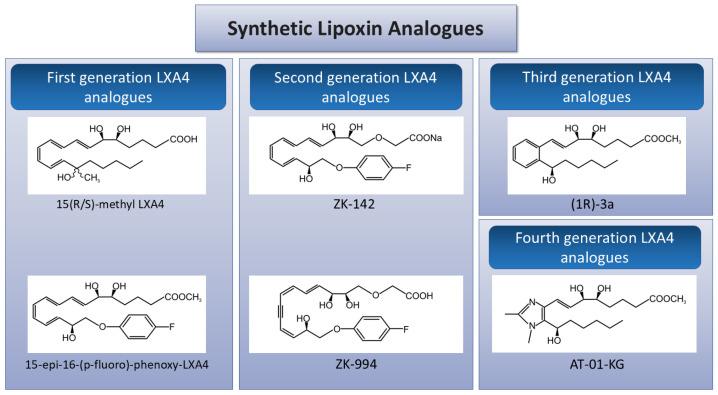
Structures of synthetic lipoxin analogs.

Second-generation lipoxin analogs (ZK-142 and ZK-994) have been developed to overcome these limitations by introducing a 3-oxa group [53], resulting in potent bioavailability after oral, topical, and intravenous administration (Figure 1). However, the complex structural elements of these analogs hinder their scalability [53]. These analogs have longer half-lives, greater potency, and enhanced anti-inflammatory activity compared to natural LXs. They show moderate selectivity for the ALX/FPR2 receptor and have demonstrated therapeutic potential in various inflammatory diseases, including asthma, rheumatoid arthritis, IBD, and periodontal disease.

Third-generation lipoxin analogs, such as (1R)-3a (Figure 1), had the modified triene core with a substituted benzo-fused ring system, simplifying the synthesis routes. These analogs demonstrated potent anti-inflammatory activity and pro-resolving actions, including agonism at ALX/FPR2 and phagocytosis of apoptotic neutrophils [54]. These compounds are engineered for precise interaction with the receptor, utilizing pharmacophoric elements that enhance binding affinity and prolong the duration of signaling. As a result, they exhibit high potency and selectivity, minimizing off-target effects. Additionally, their metabolic and chemical stability surpasses that of earlier generations, and they show promising results in conditions characterized by excessive inflammation or immune dysregulation, such as sepsis, acute respiratory distress syndrome (ARDS), Alzheimer’s disease, and cancer. While generally safe, the high potency of third-generation analogs requires careful dosing. The therapeutic efficacy of these compounds has been further validated in experimental animal models for various conditions, including renal fibrosis, obesity-related kidney and liver injury, diabetes-induced kidney disease, and atherosclerosis. RNA sequencing analysis of kidney tissues has revealed transcriptomic profiles similar to those of native lipoxins, indicating comparable protective mechanisms [55,56].

Fourth-generation imidazole- and oxazole-containing synthetic LXA4 mimetics, such as AT-01-KG (Figure 1), exhibited significant anti-inflammatory activity in vitro and in vivo. An imidazole-containing mimetic (R)-epimer of 6C-dimethylimidazole attenuated LPS-induced NF-κB activity in monocytes and inhibited neutrophil influx in zymosan-induced peritonitis [47]. It also activated calcium mobilization in an engineered ALX/FPR2 system, suggesting a potential pro-resolving capacity. In in vivo models of arthritis, AT-01-KG exerted classic anti-inflammatory effects, including retarding neutrophil influx and reducing pro-inflammatory cytokine release. Pain was attenuated and resolution was promoted by enhancing neutrophil apoptosis and efferocytosis clearance by macrophages. These findings support similar therapeutic efficacy in experimental models of inflammatory arthritis, although they have yet to be tested in renal injury and inflammation models [46].

## 5. LXs and Diseases

LXs are well-known for exhibiting anti-inflammatory effects and playing an important role in resolving various diseases. Levy et al. highlighted their involvement in resolving bronchial inflammation in asthma [57], while Wang et al. reported an association between elevated LXA4 levels and a lower risk of type 2 diabetes mellitus development [58], which is facilitated by systemic inflammation. Treatment with LXA4 reduced adipose inflammation and hepatic lipid deposition without affecting glucose tolerance [59]. The therapeutic role of LXs in kidney diseases has been linked to their ability to switch the cellular response from inflammatory to resolutory, involving the inhibition of major inflammatory events, such as the release of pro-inflammatory cytokines and accelerated clearance of recruited inflammatory cells. LXs reduced the inflammatory response associated with periodontitis [60]. LXA4 has been found to decrease the severity of endometriosis by downregulating the expression levels of ERβ, 17β-HSD1, and aromatase, thereby inhibiting estradiol production in endometriosis mouse models. Xu et al. reported that a deficiency in LXA4 may result in preeclampsia in women [61]. Consistent with this, LXA4 supplementation improved the symptoms of lipopolysaccharide-induced preeclampsia in a rat model [62]. Topical LXA4 promoted wound healing and limited the sequelae of corneal epithelial injury [63]. LX attenuated the acute phase of zymosan-induced arthritis via the modulation of ET-1 expression and its effects [64]. Saraiva-Santos et al. demonstrated that LXA4 exerts beneficial effects in a titanium dioxide (TiO_2_) arthritis model, reducing joint inflammation and pain associated with prosthesis implantation [65]. This LXA4 action was in part due to decreased TRPV1 expression and activity. LXA4 exhibited anti-carcinogenic properties, specifically against colorectal cancer (CRC) in mouse CRC cell lines [66] and reduced the proliferation of breast cancer in human and murine breast cancer cell lines [66]. The detailed roles of LXs in various diseases are described in the following sections.

### 5.1. Lung Diseases

Lung disease refers to several types of disorders that affect respiratory function. Some lung diseases are caused by bacterial, viral, or fungal infections, such as pneumonia, whereas others, including asthma, emphysema, and lung cancer, are often associated with environmental factors. Lung diseases are one of the leading causes of mortality worldwide. Below, we detail the possible roles of LXs in alleviating various lung ailments.

#### 5.1.1. Cystic Fibrosis (CF)

Cystic fibrosis (CF) is the most prevalent hereditary disease in humans. CF is caused by mutations in the gene encoding the cyclic AMP-dependent Cl^−^ channel named Cystic Fibrosis Transmembrane Conductance Regulator (CFTR) [67,68]. In the lung epithelium, tight junctions maintain cell polarity, providing a barrier against paracellular pathogen invasion, and regulate the selectivity of paracellular transport of ions and macromolecules [69]. Higgins et al. [70] reported that LXA4 protects against tight junction disruption caused by *Pseudomonas aeruginosa* (*PA*). LXA4 was shown to delay *PA* invasion and transepithelial migration in CF and standard bronchial epithelial cell cultures [70]. LXA4 prevented the reduction in mRNA biosynthesis and protein abundance of the tight junction protein ZO-1 in the bronchial epithelium. In conclusion, LXA4 plays a protective role in the bronchial epithelium by stimulating tight junction repair and reducing the invasion of *P. aeruginosa* into CF bronchial epithelial cells.

Stimulatory effects of LXA4 on ZO-1 expression and tight junction formation may constitute a crucial component of initial host defenses. Additionally, LXA4 promotes apical ATP secretion via Pannexin-1 channel and consequent P2RY11 purinoreceptor activation, increasing the airway surface liquid height and epithelial healing [71,72].

Increased neutrophil recruitment and improper neutrophil clearance are the two primary mechanisms underlying persistent airway inflammation. Recurrent infections result in neutrophil over-recruitment in CF patients’ airways. Previous studies have reported that acute inflammatory responses include an active resolution phase mediated by SPMs in mouse inflammatory exudates. LXA4 is the first eicosanoid mediator to be expressed in the active resolution phase, and it inhibits neutrophil actions [27].

**Figure 2:**
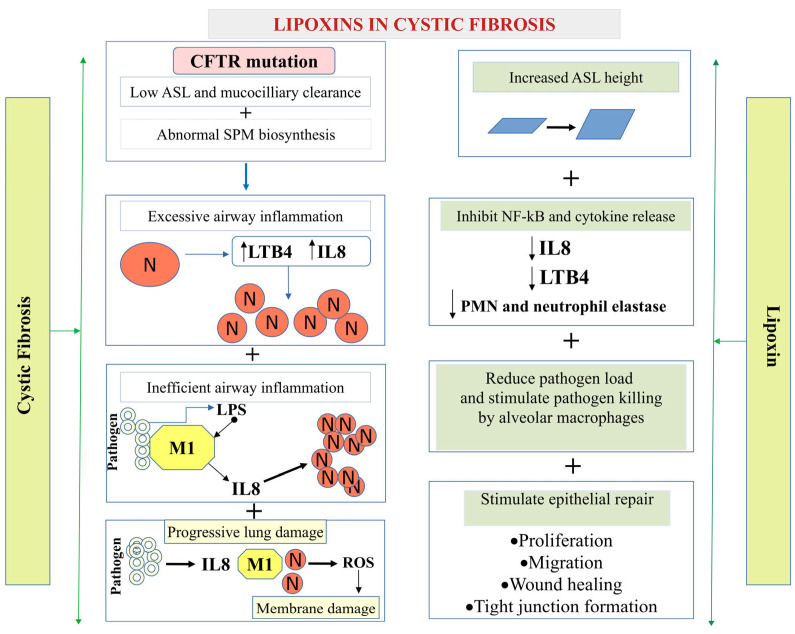
Protective effects of lipoxins against cystic fibrosis (CF) signaling pathways. Lipoxins elevate airway surface liquid height in CF bronchial epithelium and suppress the secretion of IL-8 and LTB4 in CF airway epithelial cells. Lipoxins also reduce pathogen load and stimulate epithelial repair by enhancing proliferation, migration, wound healing, and tight junction formation. “N” stands for neutrophils, and “M1” stands for inflammatory macrophages. “ROS” stands for reactive oxygen species. “↑” stands for upregulation; “↓” stands for downregulation.

LXA4 also plays an essential role in facilitating neutrophil apoptosis and stimulating the phagocytosis of apoptotic neutrophils by macrophages [73]. Previous studies have shown that delayed neutrophil apoptosis appears to be a component of the pathophysiology in patients with cystic fibrosis and is frequently correlated with disease severity and outcome. In vitro and in vivo airway studies revealed that LXA4 has potent anti-inflammatory activities. For example, LXA4 was shown to suppress IL-8 production in human airways by leukocytes and bronchial epithelial cells [74] (Figure 2).

Furthermore, it was reported that LXA4 can stimulate an intracellular Ca^2+^ mobilization in a normal human airway epithelial cell line [75]. Receptor ALX/FPR2 mediates the effect of LXA4 on airway surface liquid height as well as on calcium mobilization and Cl^−^ secretion in bronchial epithelium. The LXA4-induced increases in intracellular Ca^2+^, whole-cell Cl^−^ currents, and ASL height were inhibited by Boc-2, a specific antagonist of the ALX/FPR2 receptor. This result provides evidence for the role of LXA4 in stimulating Ca^2+^-activated Cl^−^ secretion and enhancing ASL height in non-CF and CF bronchial epithelia [76]. Hodges et al. (2017) confirmed, using specific inhibitors, that LXA4 responses were mediated via ALX/FPR2 activating phospholipases-C, -D, and -A2, which in turn stimulated the downstream signaling molecules, such as PKC, ERK 1/2, and calcium/calmodulin-dependent protein kinase (Ca^2+^/CaMK) leading to increases in [Ca^2+^]_i_ and glycoconjugate secretion [77].

#### 5.1.2. Asthma

**Figure 3:**
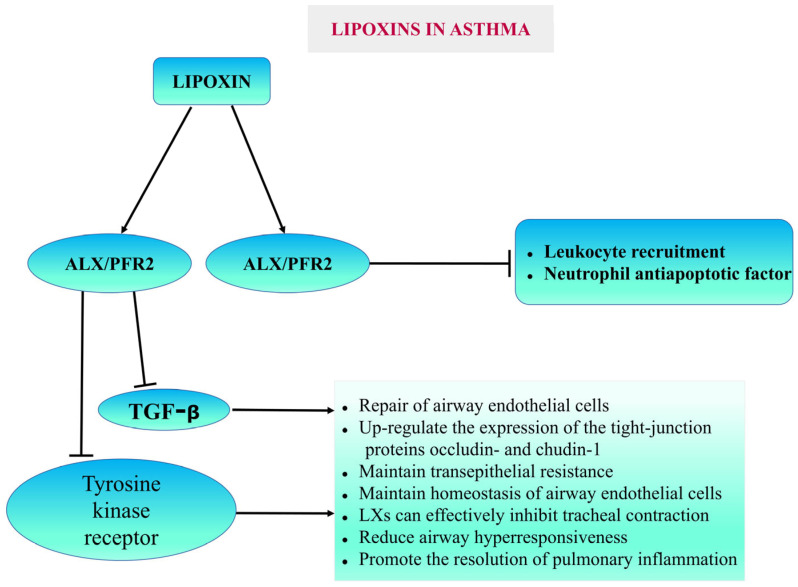
Lipoxins in asthma. Lipoxins exert protective effects against asthma by increasing the expression of ALX/FPR2, leading to inhibition of TGF-ꞵ, tyrosine kinase receptor, recruitment of leukocytes, and inhibition of neutrophil antiapoptotic factors. ”Ⱶ” stands for inhibition.

Asthma is a chronic inflammatory disorder of the airways. Asthma symptoms include airway hyperresponsiveness, reversible bronchoconstriction, and airway remodeling. LXs act as anti-inflammatory agents and protect against bronchoconstriction in asthma [78]. Previous studies have shown that LXA4 and its analogs blocked Cys-LT-mediated airway obstruction. They also inhibit LTB4-induced neutrophil and eosinophil chemotaxis. LXs have also been demonstrated to inhibit granulocyte activation and block the release of several pro-inflammatory cytokines and chemokines, including T lymphocyte cytokines, similar to the action of corticosteroids. LXs may also increase the NK cell-mediated apoptosis of both eosinophils and neutrophils [79]. Dufon et al. reported that ALX/FPR2 knockout (KO) mice exhibited a compromised resolution phenotype [40]. In asthma, patients with persistent inflammation lack ALX/FPR2 and its pro-resolution agonists. Therefore, the mechanisms that regulate ALX/FPR2 expression can be helpful as targets for enhancing endogenous anti-inflammatory responses. LXs exhibit anti-inflammatory properties, so they are reported as potential endogenous “braking signals” in the inflammatory process, and these properties indicate the potential usefulness of LXs in the treatment of asthma [80,81] (Figure 3).

#### 5.1.3. Pneumonia

Pneumonia is an infection caused by bacteria, viruses, or fungi in which the lungs’ alveoli become inflamed, filling with fluid. Pneumonia is mainly caused by *Streptococcus pneumoniae* [82] and may lead to acute respiratory distress syndrome (ARDS) and acute lung injury (ALI). LXA4 increased the survival rate of pneumonia-associated late sepsis by lowering excessive inflammatory response in pneumoseptic mice caused by *Klebsiella pneumoniae* [42]. In the early stages of sepsis, levels of the anti-inflammatory and pro-resolution mediator LXA4 and its receptor ALX/FPR2 were elevated, contributing to the dysregulation of septic inflammation [42]. Thus, LXA4 appears to exert dual effects in sepsis, with its impact depending critically on timing. Targeted modulation of the LXA4 pathways may offer a promising therapeutic strategy for sepsis management.

**Figure 4:**
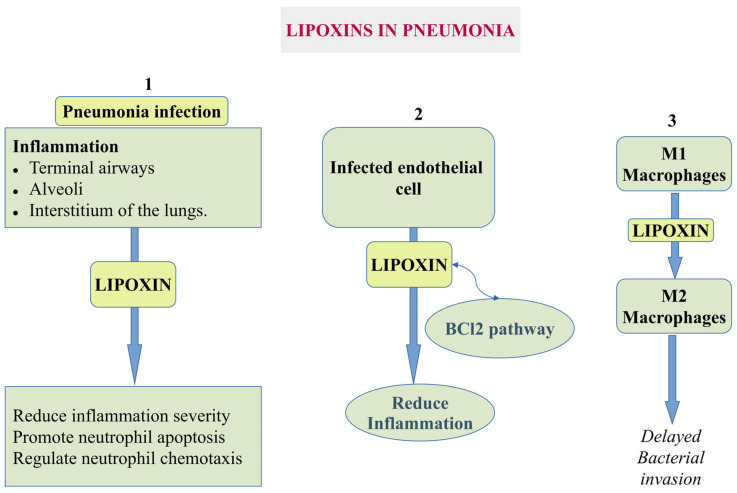
Lipoxins in pneumonia. Lipoxins mediate protection from pneumonic infection via the following mechanisms: (1) Reducing inflammation, promoting neutrophil apoptosis, and regulating neutrophil chemotaxis. (2) Targeting endothelial cells while reducing inflammation via the BCL2 pathway. (3) Reprograming macrophages and delaying bacterial invasion.

Earlier studies reported that LXA4 protects infected endothelial cells and promotes neutrophil apoptosis through the BCL2 pathway to reduce inflammation (Figure 4). Higgins and colleagues demonstrated that incubating epithelial cells with LXA4 could inhibit the infection-mediated disruption of tight junctions on the epithelial surface, thereby acting protectively and delaying bacterial invasion. Such regulation could also enhance barrier function by promoting tight junction formation and accelerating epithelial repair [70,83].

#### 5.1.4. Acute Lung Injury (ALI) and Malaria

ALI is a major feature of experimental models of severe malaria. Padua et al. reported that LXA4 exerted beneficial therapeutic effects against malaria-induced ALI by decreasing lung dysfunction, tissue injury, and neutrophil accumulation in the lungs and peripheral blood [84]. Additionally, LXA4 impaired the ability of neutrophils in *P. berghei*-infected mice to infiltrate lung tissues. The blood–brain barrier breakdown due to endothelial dysfunction is a primary feature of cerebral malaria. LXA4 ameliorated endothelial dysfunction during cerebral malaria through the regulation of ICAM-1 and HO-1 expression in brain tissues [84]. Thus, administering exogenous LXs or their analogs may limit lung tissue damage.

#### 5.1.5. Bronchopulmonary Dysplasia (BPD)

**Figure 5:**
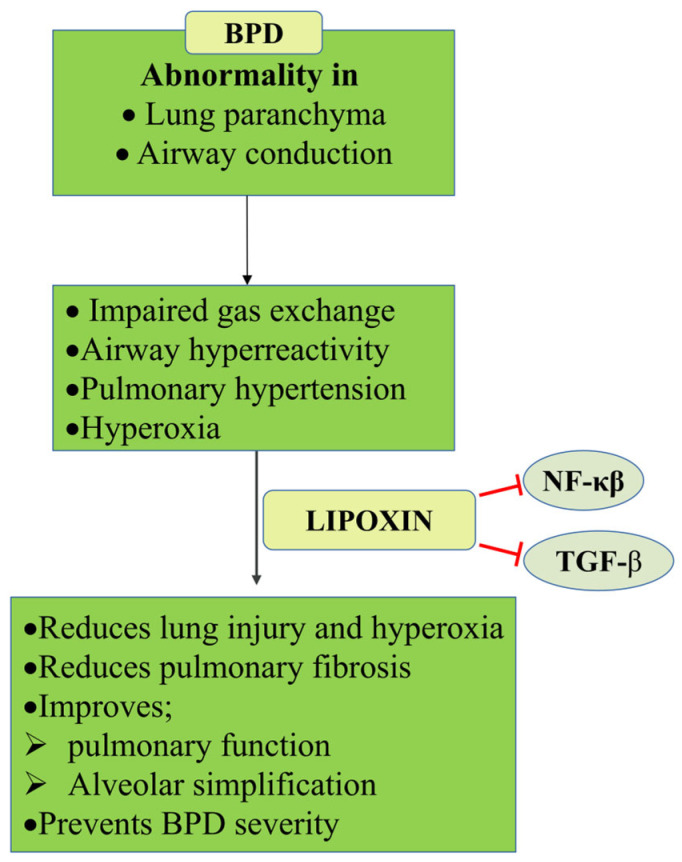
Lipoxins in bronchopulmonary dysplasia (BPD). Lipoxins exert protective effects against BPD by inhibiting TGF-ꞵ and NF-κꞵ leading to the inhibition of fibrosis and inflammation, resulting in reduced lung injury and improved pulmonary function.

BPD is common in premature infants. The main characteristics of BPD are the abnormal development of lung parenchyma, conducting airways, and pulmonary vasculature, which cause restrictions in gas exchange, airway hyperreactivity, and pulmonary hypertension, lowering physical capabilities in early childhood and later in life [85]. Previous studies demonstrated that TGF-β expression in newborn mice exposed to hyperoxia is decreased in the initial stages of BPD and increases subsequently as the disease progresses. LXs have two distinct mechanisms for controlling the TGF signaling pathway. Firstly, LXs limit the growth of NIH/3T3 and other fibroblasts by suppressing the expression of pulmonary fibrosis-related factors (such as tissue metalloproteinase inhibitor-11, matrix metalloproteinases-2 and 9, lysine oxidase-2, collagen I, elastin, and lysine oxidase-2) that may exert preventative benefits in neonatal mice with BPD brought on by hyperoxia [83,86,87] (Figure 5).

Secondly, LXA4 could significantly reduce cell and protein infiltration and oxidative stress in rat lungs, improving pulmonary function and promoting weight gain. LXA4 inhibited the release of TNF-α, MCP-1, and IL-1β in serum and BALF from hyperoxic rats [88]. The same study reported that LXA4 downregulated the expression of PINK1, Parkin, BNIP3L/Nix, and the autophagic protein LC3B. The addition of the ALX antagonist N-butyloxycarbonyl-Phe-Leu-Phe-Leu-Phe (BOC-2) partially reversed these protective effects of LXA4, indicating that LXA4 alleviates the airway inflammatory response, reduces the severity of lung injury, and improves lung function in the rat model of BPD partly through the PINK1 signaling pathway [88].

### 5.2. COVID-19

SARS-CoV-2 infection leads to the COVID-19 disease [89,90], which involves severe acute respiratory syndrome accompanied by inflammation, endothelial dysfunction, and oxidative stress [91]. In SARS-CoV-2 infection, LXs play a crucial role. Das reported in 2021 that LXs can modify SARS-CoV-2 infection by suppressing the release of pro-inflammatory cytokines, downregulating ACE2 expression, and inhibiting viral entry and replication [92].

Previous studies have reported that the insufficiency of specialized pro-resolving mediators in obese patients increases their risk of SARS-CoV-2 infection [93]. Thus, oral or intravenous LXs could effectively alleviate COVID-19 by increasing resistance and recovery from SARS-CoV-2 infection. The study also reported that LXs could act as a potential therapy against SARS-CoV-2 infection through the modulation of viral-inflammation circuits.

Endothelial dysfunction, oxidative stress, pulmonary diseases, and ALI/ARDS develop following SARS-CoV-2 infection due to persistent activation of many inflammatory pathways, such as NF-κB, STAT3, MAPK, and mTOR [94]. Initial clinical investigations showed that LX treatment may be beneficial because of its anti-inflammatory effects, which can downregulate NF-κB and STAT3 and lessen the severity of COVID-19 and associated problems [94,95]. Similarly, Cao et al. [96] reported that the lipoxin receptor agonist BML-111 inhibited the activation of the NLPR3 inflammasome in chronic obstructive pulmonary disease. Consequently, this may reduce the inflammatory alterations caused by MAPK, mTOR, and NLPR inflammasome activation [96].

**Figure 6:**
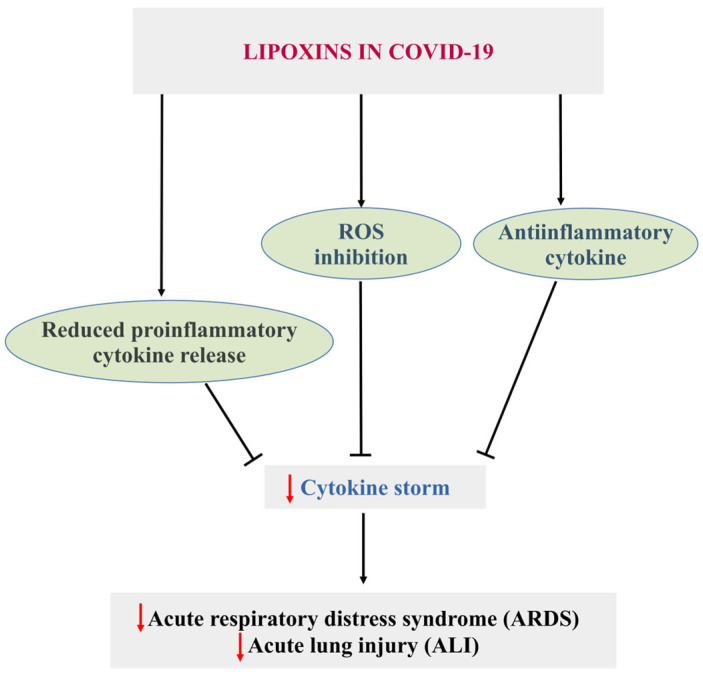
Lipoxins in COVID-19. Lipoxins decrease the cytokine storm in the late stages of COVID-19 by inhibiting ROS production, reducing pro-inflammatory cytokine release, and increasing anti-inflammatory cytokine production. These effects reduce acute lung injury (ALI) and acute respiratory distress syndrome (ARDS). “↓” stands for “decrease”; “Ⱶ” stands for inhibition.

Thus, in COVID-19 and other pulmonary diseases, LXs and synthetic LX-like agonists may block pro-inflammatory signaling pathways and stimulate anti-inflammatory cytokine production (Figure 6).

### 5.3. Cardiovascular Diseases

Cardiovascular diseases (CVDs) remain the leading cause of death worldwide, resulting in approximately 20.5 million fatalities annually (approximately 32% of all mortality worldwide) [97,98]. Several studies have demonstrated that LXs can attenuate cardiovascular pathology. Contrarily, a reduction in LXs plasma levels has been linked to the development of CVDs, confirming that LXs may play a protective role [99].

#### 5.3.1. Atherosclerosis

Atherosclerosis is a chronic disease characterized by plaque buildup in the conduit arteries. This condition is the underlying cause of various ischemic diseases, including coronary artery disease, stroke, and peripheral artery disease. Foam cell formation in atherosclerosis is driven by excessive oxidative stress, diminished antioxidant defenses in macrophages, and the uptake of oxidized LDL [100]. The prevention of macrophage to foam cell transformation is crucial for managing and protecting against atherosclerosis. LXs, specifically LXA4, are potent anti-inflammatory mediators involved in the resolution of inflammation. LXs and ATL exert their effects by binding to and activating its receptor FPR2. FPR1, FPR2, and FPR3 are members of the FPR family, and their activation leads to the phosphorylation of signaling molecules and proteins involved in NADPH oxidase activation [101,102]. ATL was found to block the progression of atherosclerosis and reduce inflammation in atherosclerosis-prone mice lacking apolipoprotein E (ApoE^−/−^ mice). ATL reduced macrophage infiltration and the number of apoptotic cells in atherosclerotic lesions, decreased the mRNA levels of inflammatory cytokines and chemokines, and showed therapeutic potential for treating atherosclerosis. Consistently, ATL had no protective effects against atherosclerosis in ApoE^−/−^ mice lacking the FPR2 receptor [103]. Conversely, elevated expression of ALX/FPR2 was found in human carotid atherosclerotic lesions on macrophages, smooth muscle, and endothelial cells within the atheromas [38]. The same authors found that the volume of advanced carotid atherosclerotic lesions and pro-inflammatory chemokine and cytokine production in leucocytes correlated positively with the relative ALX/FPR2 expression levels. Interestingly, these carotid lesions exhibited an increased plaque stability, likely due to the enhanced smooth muscle cell migration and proliferation leading to the formation of a protective fibrous cap [38]. Consistently, Petri et al. found that atherosclerosis progression was slowed down in Ldlr^−/−^;FPR2^−/−^ mice compared to Ldlr^−/−^ mice and accelerated in Ldlr^−/−^;FPR2^−/−^ mice after a Ldlr^−/−^;FPR2^+/+^ mouse bone marrow transplantation, highlighting the involvement of ALX/FPR2-mediated pro-inflammatory signaling in atheroma-associated macrophages. The author concluded that ALX/FPR2 signaling may play a dual role during atherosclerosis by (i) promoting disease progression and (ii) increasing plaque stability while facilitating fibrous cap formation. The dual role of the FPR2 receptor in atherosclerosis could be attributed to the cellular environment and ligand-biased behavior of the receptor. The specific cell type (macrophage, smooth muscle cell, or endothelial cell) and the prevailing plaque microenvironment dictate how FPR2 activation translates into pro- or anti-atherosclerotic outcomes. Within macrophages, FPR2 engagement by pro-inflammatory ligands can critically perpetuate chronic inflammation and accelerate foam cell formation, contributing to disease progression [38]. However, the very same receptor, when activated by pro-resolving agonists like ATL on vascular smooth muscle cells, actively contributes to plaque stability by facilitating fibrous cap formation [103]. Distinct ligands, such as pro-inflammatory mediators and pro-resolving mediators, bind to FPR2 and induce different conformational changes in the receptor, leading to the activation of distinct downstream signaling pathways. Different ligands stabilize different active conformations of the receptor, which then couple to different sets of intracellular signaling proteins and give different responses [104].

Later, Kraft et al. demonstrated in a cohort of patients with atherosclerosis that LXs can reduce excessive peripheral neutrophil ROS production, attenuate the upregulation of clot-activating integrin CD11b, and enhance lymphatic neutrophil migration. These effects were specific to neutrophils isolated from patients with atherosclerosis and depended on the individual’s inflammatory status. The authors indicated that although lipoxin treatment has potential as a therapeutic approach for atherosclerosis, its effectiveness may vary based on the patient’s inflammatory condition [37]. The authors also found that LXB4 displayed more potent effects on neutrophil function compared to LXA4, indicating that there may be some variability in specific LX subtype ability to modulate atherosclerosis progression.

Using a rabbit model of high-fat-diet-induced atherosclerosis, Mai et al. [105] found that LXA4 exhibited an atheroprotective effect by inhibiting foam cell formation, oxLDL-induced inflammation, and apoptotic signaling in macrophages. The authors employed THP-1 macrophages and human monocyte-derived macrophages to investigate the underlying mechanisms. They demonstrated that LXA4 treatment decreased the protein expression levels of CD36 and SR-A, which are involved in cholesterol uptake. This LXA4 effect was likely mediated through the FPR2 receptor activation because BOC-2, a LXA4 receptor FPR2 antagonist inhibited it. Additionally, the authors showed that LXA4 inhibited macrophage apoptosis, likely by decreasing the oxLDL-induced activation of the c-Jun N-terminal kinase pathway and inhibiting caspase-3 activation.

BML-111, a synthetic analog of LXs, was also reported to attenuate atherosclerosis progression in rats fed a high-fat diet by activating NF-E2-related factor 2 (Nrf2) signaling. Nrf2 is a transcription factor that plays a central role in regulating the expression of antioxidant and detoxifying genes, thereby protecting cells from oxidative stress. BML-111 exerted its beneficial effects by modulating Keap1/Nrf2, which is translocated into the nucleus, leading to increased gene expression involved in antioxidant defense. In both in vivo and in vitro experiments, LXA4 inhibited the transformation of macrophages into foam cells by activating Nrf2 signaling. This effect was accompanied by decreased levels of oxidative stress markers, such as malondialdehyde (MDA), and improved lipid profiles, including increased high-density lipoprotein levels, but was independent of the LXA4 receptor (formyl peptide receptor 2). BML-111 was found to decrease inflammatory mediators, such as IL-1β, MCP-1, IL-6, VCAM, ICAM, and TNF-α, confirming the molecule’s anti-inflammatory properties [106].

Thus, LXs show great potential in preclinical studies by inhibiting inflammation, foam cell formation, and promoting plaque stability. However, further research is needed to elucidate how different LXs and their receptors contribute to slowing down atherogenesis. Current lipoxin-based therapies for atherosclerosis are still in the experimental stage, and no FDA-approved drugs are available. Furthermore, LXs’ short half-life and rapid metabolism pose challenges. Lipoxin synthetic analogs may help overcome these limitations, but clinical trials are still needed to evaluate their efficacy and safety.

Remarkably, low-dose aspirin, a classical antithrombotic medication, is routinely prescribed to cardiovascular patients to prevent clot formation and reduce the risk of heart attacks and strokes, and an estimated number of 29 million persons are taking aspirin daily for the prevention of cardiovascular disease [107]. However, it remains to be determined whether well-known aspirin’s anti-inflammatory effects are at least partly due to the production of aspirin-triggered lipoxin A4.

#### 5.3.2. Abdominal Aortic Aneurysm

Abdominal aortic aneurysm (AAA) is a condition characterized by the weakening and bulging of the aortic wall in the abdominal section of the aorta. Large AAAs are prone to rupture, which is a clinical emergency and often fatal. Inflammation plays an important role in the development of an aortic aneurysm [108]. Decreased expression of ALX/FPR2 in AAA lesions was associated with increased inflammation and progression of the disease. Petri et al. reported that lipoxin formation and LXA4 formyl peptide receptor 2 expression are important for resolving inflammation and preventing abdominal aortic aneurysm progression, localized enlargement, or bulging [109].

#### 5.3.3. Cardiomyopathy

Cardiomyopathy is a group of diseases that affect the heart muscle (myocardium), impairing its ability to pump blood effectively. Researchers have developed an animal model of experimental autoimmune myocarditis (EAM) and treated mice with BML-111. They found that BML-111 treatment reduced immune cell infiltration and decreased pro-inflammatory mediator levels in the heart. Additionally, BML-111 improved cardiac function in EAM mice by reducing cardiomyocyte apoptosis, reversing harmful changes in heart size and fibrosis, and restoring proper cell contraction. These effects were achieved by reducing oxidative stress by activating the NRF2 antioxidant response via the CaMKK2/AMPKα signaling pathway [110]. In agreement, analysis of blood plasma samples from patients with dilated cardiomyopathy (DCM) revealed that reduced plasma LXA4 levels strongly correlated with the severity of the disease [111].

LXA4 protects against EAM by reducing inflammation and inhibiting the NF-B and PI3K/Akt signaling pathways. These mechanisms contribute to the attenuation of the inflammatory response and prevent further myocardial damage in the context of autoimmune myocarditis [112]. Lipoxins mitigated lipotoxicity and inflammation associated with DCD. They promoted inflammation resolution by upregulating the expression of peroxisome proliferator-activated receptor gamma (PPARγ) and by regulating CD36, a fatty acid translocase and scavenger receptor [113]. PPARγ plays a significant role in lipid and glucose homeostasis through its involvement in the sterol regulatory element-binding protein (SREBP) signaling pathway.

Preclinical studies have shown promising results for the effectiveness of LXs and their synthetic analogs, which can attenuate cardiac inflammation, reduce oxidative stress, improve myocardial function, and promote tissue repair in experimental cardiomyopathy models. These findings suggest LXs as a promising therapeutic option for treating cardiomyopathy.

#### 5.3.4. Myocardial Infarction

Myocardial infarction, commonly known as a heart attack, occurs when there is a blockage in one or more of the coronary arteries, which supply oxygen-rich blood to the myocardium. Patients with acute myocardial infarction (AMI) who had higher plasma LXA4 levels showed a decreased risk of major adverse cardiovascular events. Notably, individuals with elevated LXA4 levels and low hsCRP levels (an inflammation marker) had the lowest risk of myocardial ischemia [114]. Kain et al. demonstrated the important role of 15-epi LXA4 in the inflammation resolution phase following a heart attack. 15-epi LXA4 promoted the clearance of immune cells and activated FPR2 and GPR120 on macrophages while inhibiting the GPR40 receptor. The authors found that administering 15-epi LXA4 in liposomes or in free form after a heart attack improved heart function and reduced inflammation [115].

Cardiac fibrosis is a pathological feature in patients with myocardial infarction. Lipoxins and their analog BML-111 exert therapeutic effects against cardiac fibrosis by activating Nrf2 [116]. The risk of myocardial infarction and stroke may potentially be increased in patients treated with selective COX-2 inhibitors, in part because these drugs may inhibit the formation of ATL [117]. However, treatment with aspirin, an unspecific and irreversible inhibitor of COX-1 and COX-2 enzymes, enhances ATL formation. This is because aspirin-treated COX-2 enzymes produce ATLs. LXs and ATLs attenuated inflammation, reduced infarct size, and improved cardiac function in animal myocardial infarction models. LXs and ATLs also have the potential to promote tissue repair. Conversely, selective COX-2 inhibitors interfere with the formation of ATLs and increase the risk of myocardial infarction [118]. Indeed, clinical studies have provided evidence that low-dose aspirin is beneficial for preventing myocardial infarction [119]. However, further large-scale randomized clinical studies will be needed to establish whether LXs, ATLs, or their synthetic analogs are cardioprotective in clinics.

#### 5.3.5. Other Thrombotic Vascular Disorder

Ischemic stroke is a type of stroke that occurs when the blood supply to a part of the brain is interrupted or severely reduced, leading to damage or death of central neurons. Preclinical studies have demonstrated that BML-111 significantly reduces stroke size and protects the cerebral cortex. This effect is likely achieved by diminishing the blood–brain barrier’s permeability and suppressing chemokine and pro-inflammatory cytokine synthesis, including tumor necrosis factor and interleukins (IL-1, IL-6, and IL-8), which helps preserve the integrity of brain tissue [120].

However, the development of lipoxin-based therapeutics for ischemic stroke is still in its early stages. Preclinical studies have demonstrated that lipoxin can attenuate inflammation, reduce brain damage, and improve functional outcomes in animal models of ischemic stroke. These findings highlight the potential of targeting the LX pathway as a therapeutic strategy for ischemic stroke [121].

**Figure 7:**
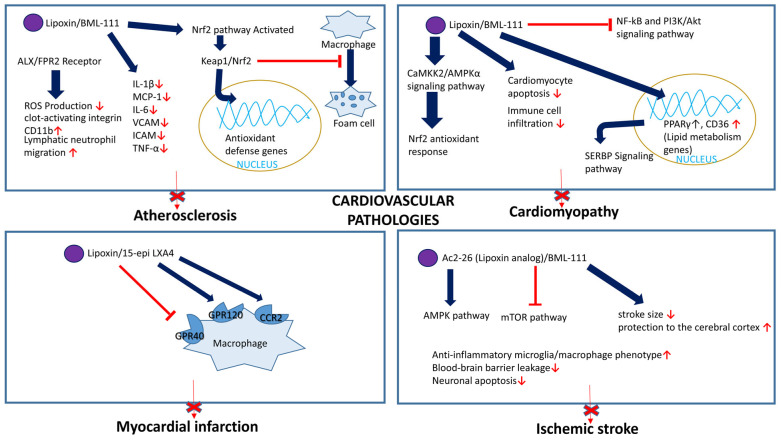
Lipoxins and the cardiovascular system. The role of lipoxin and its synthetic analogs in the prevention of various cardiovascular diseases. The **upper left** panel shows lipoxin-induced atheroprotective changes in the vascular wall during atherosclerosis progression. The **upper right** panel shows lipoxin-induced cellular changes in cardiomyocytes and immune cells, leading to reduced cardiomyopathy. The **lower left** panel shows the effect of lipoxins on macrophages, resulting in reduced myocardial infarct volume. The **lower right** panel shows the effects of lipoxin analogs leading to reduced stroke size and cerebral cortex protection. “↑” stands for upregulation; “↓” stands for downregulation; ”Ⱶ” stands for inhibition.

In summary, LXs are promising therapeutic agents for cardiovascular diseases because of their anti-inflammatory, pro-resolving, and tissue-protective properties. LXs and their synthetic analogs interfere with various signaling cascades and hence are important in preventing multiple cardiovascular diseases (Figure 7). However, further research is needed to translate these findings into clinically effective treatments that improve outcomes and the management of cardiovascular conditions.

### 5.4. Microbial Infection

**Figure 8:**
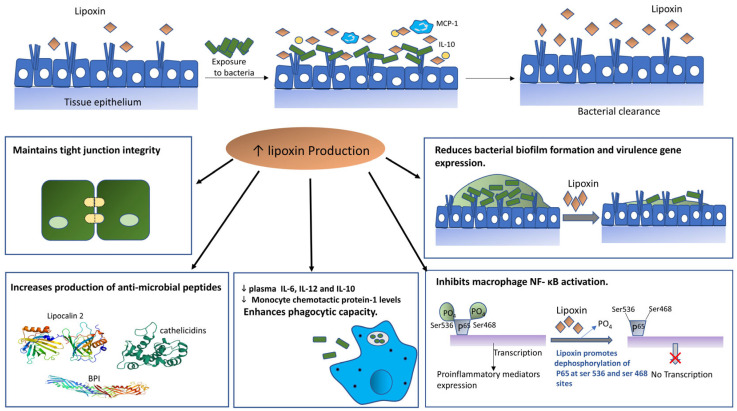
Lipoxins and bacterial infections. Lipoxins maintain the integrity of tight junctions between enterocytes in the small intestine, preventing pathogen infiltration from the intestinal lumen into the bloodstream (the gut-blood barrier). Lipoxins increase the production of antimicrobial peptides, such as lipocalin 2, cathelicidin, and BPI. Lipoxins also enhance the phagocytic capacity of macrophages and reduce their release of chemo- and cytokines, MCP-1, IL-6, IL-10, and IL-12. Lipoxins reduce bacterial biofilm formation and inhibit macrophages’ NF-κB activation.

LXA4 is an endogenous inflammation-resolving factor. However, host defense against bacterial infection requires inflammation. This section discusses the role of LXs in diseases caused by various microbial infections (Figure 8).

#### 5.4.1. *Staphylococcus aureus*

Septic arthritis is an infectious articular disease associated with high morbidity and mortality among patients [122,123]. The main challenge in patients with septic arthritis is controlling bacterial replication while preventing or decreasing the articular damage caused by *Staphylococcus aureus* infection. Boff et al. (2020) demonstrated that 5-LOX blockade diminished joint inflammation and articular tissue damage [124]. Additionally, *S. aureus* in the joint was better controlled when 5-LOX was genetically or pharmacologically blocked. Boff et al. concluded that inhibiting LXA4 synthesis or the LXA4 receptor can lead to better outcomes in a model of *S. aureus*-induced arthritis.

#### 5.4.2. *Klebsiella pneumoniae*

As indicated above, LXA4 has a substantial anti-inflammatory property, but the host inflammatory response is necessary to reduce bacterial infection. In sepsis, LXA4 may play a dualistic role. Sordi et al. (2013) demonstrated the role of LXA4 and its receptor ALX/FPR2 in the dysregulated inflammatory response during sepsis [42]. Pneumosepsis was induced in mice via *Klebsiella pneumoniae* inoculation. The study assessed plasma LXA4 levels and ALX/FPR2 receptor expression throughout the infection and evaluated the effects of receptor agonists (LXA4 and BML-111) and antagonists (BOC-2 and WRW-4) administered at early (1 h) and late (24 h) stages of sepsis [42].

It was found that the levels of LXA4 were increased at the onset of pulmonary sepsis, and this early increase seemed to contribute to the inappropriate response of the host to the infection [42]. In the early phase of sepsis, the immune system initiates a strong inflammatory response triggered by pathogen-associated molecular patterns (PAMPs) that activate toll-like receptors (TLRs). This activation increases the production of pro-inflammatory cytokines, such as TNF-α and IL-1β, which are essential for recruiting neutrophils to clear the pathogens. Simultaneously, LXA4, an anti-inflammatory lipid mediator, rises and binds to its receptor ALX/FPR2, located on macrophages and immature myeloid cells. The increase in LXA4 levels suppresses both neutrophil recruitment and cytokine production, potentially hindering the body’s ability to control the infection and worsening outcomes. In the late phase of sepsis, the immune system transitions to a compensatory anti-inflammatory state characterized by immune cell exhaustion and chronic cytokine release, which can damage organs. In this phase, LXA4 plays a protective role by reducing systemic inflammation, improving organ function, and enhancing survival.

Thus, these findings reveal the detrimental role of LXA4 in early sepsis and suggest that pharmacological inhibition of the ALX/FPR2 receptor may favorably affect the survival of animals presenting with pneumosepsis. However, the administration of LXA4 in late sepsis may be beneficial because it reduces the excessive inflammatory response and improves survival.

#### 5.4.3. *Borrelia burgdorferi*

*Borrelia burgdorferi* is a tick-borne spirochete responsible for developing Lyme arthritis. It was reported that 5-LOX metabolites play a role in decreasing inflammation during this infection. It was found that the severity of arthritis increased and persisted when 5-LOX was absent [125]. Furthermore, in vitro research has shown that macrophages from C3H 5-lipoxygenase (5-LOX)^−/−^ mice exhibited impaired phagocytosis of *Borrelia burgdorferi*; however, leukotriene B4 (LTB4), another 5-LOX metabolite, promoted the phagocytosis of *Borrelia burgdorferi* via BLT1 or BLT2 receptors [126].

#### 5.4.4. *Pseudomonas aeruginosa*

*Pseudomonas aeruginosa* lung infection, which eventually impairs lung function, is a defining characteristic of cystic fibrosis. According to recent research, the loss of CFTR inhibits lipoxin production, thereby preventing the healing of lung inflammation and promoting the spread of new infections [127]. Higgins et al. described the protective effect of LXA4 (1 nM) against tight junction disruption caused by a bacterial challenge with *Pseudomonas aeruginosa* and the delayed action against bacterial invasion in cystic fibrosis airway epithelial cells [70].

#### 5.4.5. *Cryptococcus neoformans*

Colby et al. (2016) studied strain-specific differences in *Cryptococcus neoformans* (*Cne*) clearance due to variations in immune responses, pro-resolving LXA4 biosynthesis, and ALX/FPR2 receptor expression [36]. Strain-dependent differences in the reduction in Cne burden became apparent 14 days after lung infection. C57BL/6 mice did not effectively lower lung *Cne*, which persisted and increased over a 28-day interval. LXA4 levels were elevated in C57BL/6 mice compared with CB-17 mice, suggesting strain-dependent differences in Alox12/15 activity. The expression of the LXA4 and 15-epi-LXA4 receptor ALX/FPR2 was decreased in C57BL/6 mice compared to CB-17 and was associated with Th2-type lung inflammation and decreased capacity to reduce fungal burden. In sharp contrast, CB-17 mice exhibited a decreased lung *Cne* infection with a more robust Th1 response and cytokine (IFN-g and IL-17) production associated with inducible ALX/FPR2 expression and regulation of pathogen-mediated inflammation by 15-epi-LXA4. These findings suggest that SPMs, such as LXs, play crucial roles in regulating fungal host defense in the lung [36].

#### 5.4.6. *Toxoplasma gondii*

LXs regulate the immune response to *Toxoplasma gondii* infection [128]. It was found that serum lipoxin A4 levels increased during infection in a mouse model of *T. gondii* infection (established through intraperitoneal inoculation of *T. gondii* cysts) and remained high if chronic disease had been confirmed [129]. Compared with wild-type control mice, 5-lipoxygenase^−/−^ transgenic mice that cannot synthesize lipoxin A4 had increased serum levels of IL-12 and IFN-γ and exhibited higher infection-related mortality rates, but fewer *T. gondii* brain cysts and a lower parasite load [130]. Therefore, despite better parasite control, it seems likely that the increased mortality of lipoxin-deficient mice is due to tissue damage caused by cytokines. Corroborating this finding, the histological severity of meningitis and encephalitis was increased in transgenic mice. The administration of a stable LXA4 analog (15-epi-16-phenoxy-parafluoro-LXA4-methyl ester, 0.25 mg/kg) rescued lipoxin-deficient mice from this fatal phenotype in part by decreasing IL-12 and IFN-γ levels [130]. Remarkably, treatment with IL-10 (0.1 μg/animal) in the same *T. gondii*-infected lipoxin-deficient mice was much less effective.

Thus, interleukin-12, produced by antigen-presenting cells such as dendritic cells, plays a vital role in host control of intracellular pathogens such as *T. gondii* and viruses, but its excess can be fatal. In mice, LXA4 analogs suppress IL-12 production in dendritic cells stimulated with *T. gondii* extract by acting on the AhR and LXA receptors. The activation of these two receptors triggered an increased expression of a suppressor of cytokine signaling 2 (SOCS-2), which inhibited IL-12 production in dendritic cells [129]. Although this may be a host-driven response to curb excessive inflammation, the induction of lipoxin production could also be a strategy adopted by the pathogens to modulate host immunity and perhaps facilitate chronic infection by reducing tissue damage [130,131,132].

#### 5.4.7. *Trypanosoma cruzi*

Chagas disease (American trypanosomiasis) is a chronic condition caused by the parasite *T. cruzi*. It was reported that in mice infected with *T. cruzi*, administration of aspirin (acetylsalicylic acid) at 25 or 50 mg/kg improved survival rates, reduced heart damage, and reduced the highest parasite infection levels [133]. However, higher or lower aspirin doses did not have any effect. The study also found that aspirin increased the production of 15-epi-lipoxin A4 in an in vitro setting and live infection models. Additionally, the infection itself increased the levels of 15-epi-lipoxin A4. External administration of 15-epi-lipoxin A4 also decreased parasitemia, reduced inflammation in the heart, and improved survival rates. Based on these findings, it was proposed that the beneficial effects of aspirin in infected mice were attributable to the production of 15-epi-lipoxin A4. This discovery could provide new therapeutic strategies for treating Chagas disease during its acute phase [133].

#### 5.4.8. *Porphyromonas gingivalis*

Gram-negative bacterium *P. gingivalis* is the main cause of periodontal diseases. *P. gingivalis* bacteria form biofilms on the tooth surface and lead to gum disease [134]. Serhan et al. used a rabbit model of periodontitis to explore the impact of LXs [135]. Periodontitis in wild-type rabbits was induced by applying a dental ligature along with *P. gingivalis*, resulting in tissue loss, histological changes (infiltration of white blood cells), and radiological signs of bone loss. The researchers then utilized transgenic rabbits that had increased production of LXs due to overexpression of the enzyme 15-lipoxygenase. Interestingly, when these transgenic rabbits underwent the same procedure to induce periodontitis, they did not exhibit any clinical, histological, or radiographic features associated with the condition. Additionally, the team demonstrated that intravenous metronidazole administration prevented bone loss in wild-type rabbits presenting with periodontitis, confirming that infection by *P. gingivalis* was responsible for the observed effects [135]. Furthermore, the authors showed that a topical lipoxin analog treatment efficiently protected rabbits presenting with periodontitis from significant tissue damage, accumulation of white blood cells, and bone loss. While periodontitis is an infectious disease, the tissue damage characteristic of this condition is believed to be driven by an abnormal response of the host to the infection. The series of experiments conducted by Serhan et al. revealed that LXs may play a role in suppressing this excessive response, thereby offering a protective effect in vivo [135].

### 5.5. LXs in Sepsis

Sepsis is a severe dysregulated immune response that may occur during a bacterial infection or sometimes a parasitic infection. Sepsis is associated with marked elevation of plasma levels of cytokines, such as IL-1β, IL-6, interferon γ, and TNFα. In bacterial sepsis, bacterial wall components stimulate a strong immune response by activating TLR2 or TLR4 receptors on immune cells. Specifically, lipopolysaccharide (LPS) from the wall of Gram-negative bacteria activates TLR4 receptors, whereas peptidoglycan from Gram-positive bacteria stimulates TLR2 receptors [136]. Parasitic infection is associated with the activation of either TLR or the high-affinity IgE receptor (Fc epsilon RI) [137,138]. We noted in the previous chapters that the role of LXs in sepsis is stage-dependent and may lead to beneficial or detrimental effects [42].

It was reported that the excessive pro-inflammatory response in a sepsis model associated with *Toxoplasma gondii* infection was reduced by LXA4 [128]. Similarly, Wu et al. reported that in vivo LXA4 treatment in a clinically relevant sepsis model of the cecal ligation and puncture promoted morphological changes in peritoneal neutrophils linked to increased bacterial clearance [139]. Decreased cell migration, enhanced apoptosis, and augmented phagocytic capacity without an increase in free radical production were the characteristics of these changes. Achieving this phenotype is advantageous because it reduces the sepsis-induced neutrophil lifespan while improving phagocytic activity without producing excessive free radicals [139]. Conversely, LXA4-mediated immune suppression may weaken the Th1 responses, promoting bacterial persistence, as occurs in a model of Mycobacterium tuberculosis infection [140] which involves TLR-2 activation [141]. Therefore, inhibiting LX effects can increase host resistance to Mycobacterium tuberculosis [140]. Thus, diverse pathogens can engage distinct immune pathways, and LXs may exhibit either protective or detrimental effects depending on the type of pathogen involved.

### 5.6. Stem Cells

The significance of LXs in stem cell biology includes many key aspects, such as promoting anti-inflammatory phenotypes, enhanced stem cell migration and homing, and modulation of stem cell differentiation. LXs also protect against oxidative stress and regulate immune responses while promoting tissue repair and regeneration. For example, LXA4 plays a significant role in boosting the regenerative potential of periodontal ligament stem cells (PDLSCs). It was discovered that LXA4 promotes the proliferation, migration, and osteogenic differentiation of PDLSCs in vitro and enhances their impaired osteogenic capacity in vivo. Specifically, the PI3K/AKT pathway was identified to be critical for mediating the effect of LXA4 on the osteogenesis of inflammatory PDLSCs [9,142,143].

The LXA4-ALX/FPR2 axis also plays a role in regulating the Stem Cells of the Apical Papilla (SCAP) function. Gaudin et al. reported that ALX/FPR2 expression was upregulated after stimulation with lipopolysaccharide or TNF-α [9] in SCAP. Notably, LXA4 enhanced the wound healing capacity of SCAP and inhibited cytokine, chemokine, and growth factor secretion by SCAP in an ALX/FPR2-dependent manner. These features support the role of these cells in the resolution phase of inflammation and suggest a novel molecular role for the ALX/FPR2 receptor in enhancing stem cell-mediated pro-resolving pathways.

A compelling argument was made that LXs and leukotrienes could play crucial roles in human myelopoiesis. By analyzing the modulatory effects of these compounds on GM-CSF-induced myeloid stem cell proliferation, leukotrienes and LXs might be involved in regulating the production of blood cells in the bone marrow [144,145,146,147,148].

Another significant aspect of LXs’ interaction with stem cells involves their role in modulating immune system responses. LXs may be vital as autacoids in controlling inflammation and orchestrating its resolution. Likely, LXs serve as “stop signals” for inflammation, effectively guiding cellular responses toward non-phlogistic monocyte recruitment, which leads to the resolution of the inflammatory response or promotes repair and healing [149].

It was reported that a combination of the FPR2 agonist, LXA4, and exosomes derived from human umbilical cord mesenchymal stem cells effectively controlled inflammation and promoted tissue repair in the context of preterm premature rupture of membranes (pPROM), a prevalent obstetric complication [150]. Further emphasizing the importance of LXs in tissue repair, studies have shown that human amnion epithelial cells (hAECs)-derived LXA4 has immunomodulatory properties and contributes to the communication between hAECs, macrophages, T cells, and neutrophils during tissue repair and inflammation resolution [151,152,153].

Mesenchymal stem cell (MSC) treatment is effective in Diabetic Neuropathy (DN) models by protecting renal function and preventing fibrosis. A study demonstrated that MSC intervention prevented DN progression via the LXA4-ALX/FPR2 axis, which inhibited glomerulosclerosis and pro-inflammatory cytokines, eventually contributing to kidney homeostasis [154].

Apart from regulating acute inflammatory responses, leukotrienes and LXs may help restore stem cells by directly regulating the proliferation and differentiation of neuronal stem cells [155].

LXA4 likely has a time- and dose-dependent effect on embryo implantation because the preimplantation administration of LXA4 resulted in implantation failure. It was demonstrated that the most effective time to use LXA4 to block embryo implantation was on Day 0.5 after fertilization [156]. It was speculated that, in part, the effect was due to the ability of LXA4 to inhibit epithelial–mesenchymal transition.

**Figure 9:**
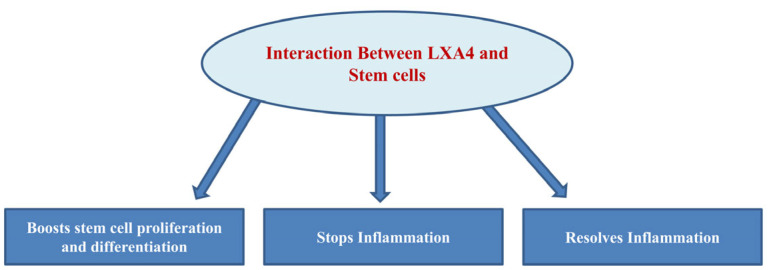
Lipoxins increased the rate of stem cell proliferation and differentiation leading to reduced inflammation and facilitated inflammation resolution.

The interaction between LXs and stem cells offers promising avenues for future research and therapeutic applications (Figure 9). From boosting stem cell proliferation and differentiation to controlling inflammation and promoting tissue repair, the role of LXs appears pivotal across various biological phenomena and disease conditions. Notably, aspirin-triggered LX production mimics the bioactivation of native LXs in several biological systems, including stem cells [6]. Overall, the role of LXs in modulating stem cell function has the potential to enhance the efficacy and outcomes of regenerative medicine. However, further studies are needed to develop clinically effective LX-based therapies.

### 5.7. Diabetes

The likelihood that a person will develop chronic inflammatory disorders, such as accelerated atherosclerosis or inflammatory bowel disease, is increased in diabetic individuals, in part, owing to impaired resolution of inflammation. The synthesis of endogenous modulators of inflammation, such as LXA4, is critical for resolving inflammation [11,55]. Studies have demonstrated that chronic inflammatory conditions can progress due to the inability to control inflammation. Thus, treating chronic inflammatory disorders, such as diabetes complications, could be achieved by focusing on the resolution of inflammation.

LXs reduce inflammation by inhibiting neutrophil invasion, promoting macrophage polarization, increasing macrophage efferocytosis, and restoring tissue homeostasis [157]. Research has shown that LXs and their synthetic analogs shield tissues from acute and chronic inflammation by downregulating IL-1 and TNF-α production along with inhibiting the nuclear factor light chain enhancer of activated B cells (NF-κB) pathway and increasing the release of pro-resolving cytokines like interleukin-10 [11].

Cellular and animal studies demonstrated that LXA4 may directly affect the adipocyte insulin signaling pathway, potentially delaying the development of type 2 diabetes mellitus (T2DM) [58]. This may be the mechanism by which LXA4 helps control diabetes. Elias et al. (2016) reported that treatment with LXA4 in adipose tissue explants from aging mice—a model of fatty inflammation—leads to a decrease in IL-6 and restoration of GLUT4 and insulin receptor substrate 1 (IRS1) expression, indicating less inflammation and improved insulin sensitivity [158]. It has also been found that treatment of mice with LXA4 protects against high-fat-diet (HFD)-induced adipose tissue inflammation and hepatic lipid deposition without affecting glucose tolerance [59].

**Figure 10:**
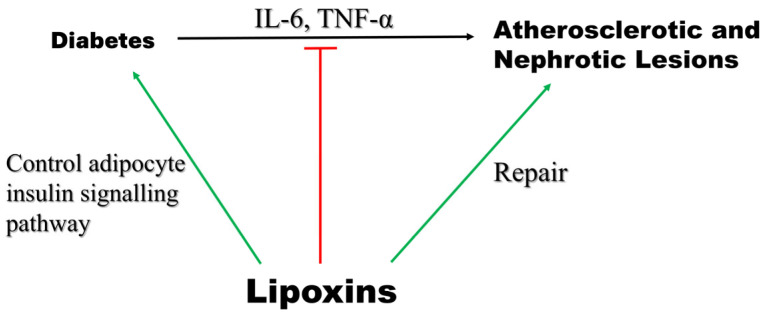
Lipoxins and diabetes. During diabetes, the elevated expression levels of IL-6 and TNF-α promote atherosclerosis and kidney damage. Lipoxins inhibit IL6 and TNF-α production in diabetes and control adipocyte insulin signaling pathways. Lipoxins also promote repair processes that may lead to atherosclerosis regression. A hallmark of diabetic heart failure is accelerated glycation and altered lipid metabolism. SREBP signaling significantly influences the homeostasis of lipids and glucose. Therefore, by inhibiting the SREBP signaling, AGEs worsen diabetic cardiomyopathy’s lipotoxicity. LXs reduce lipotoxicity-induced inflammation by increasing PPARϒ expression and controlling CD36. In SREBP signaling, PPARϒ mediates the link between glycation and LXs [113]. ”→ ” stands for activation; ”Ⱶ” stands for inhibition.

Pro-inflammatory cytokines, including IL-6 and TNF-α, and ROS production are increased in both type 1 and type 2 diabetes mellitus, although the degree may be higher in type 1 diabetes mellitus. Such upregulation of pro-inflammatory cytokines and ROS may promote peripheral insulin resistance and cell apoptosis. The mechanisms causing such alterations are thus far poorly understood. However, it has been reported that animals with induced type 1 diabetes mellitus and people presenting with type 2 diabetes mellitus exhibit low plasma concentrations of arachidonic acid (AA) and lipoxin A4 (LXA4) [159]. Notably, LXA4 blocks the synthesis of IL-6 and TNF-α and reduces ROS generation [159]. Thus, LXA4 and LXB4 may function as endogenous antidiabetic molecules, implying that their administration could be helpful in the prevention and management of both types of diabetes mellitus [160] (Figure 10). LXA4, along with BDNF (brain-derived neurotrophic factor), EPA, DHA, AA, and GLA (gamma-linolenic acid), has been proposed to protect pancreatic β cells from the cytotoxic action of various chemicals, such as alloxan (AL), streptozotocin (STZ), doxorubicin (DB), and benzo(a)pyrene (BP)-induced cytotoxicity and may prevent the development of diabetes mellitus [161].

Poor bioavailability, high instability, and lower water solubility significantly complicate the potential clinical applications of LXs. Taking supplements containing arachidonic acid during inflammatory processes may lead to both positive and negative outcomes. Furthermore, although such supplements may boost the synthesis of healthy LXs, they can also raise pro-inflammatory mediator production, such as prostaglandins and leukotrienes, which may make it more difficult to resolve inflammation [6,162].

In animal models, LXA4 reversed adipose tissue autophagy triggered by a high-fat diet [158], suggesting LXs can be utilized as therapeutics for obesity-induced diseases. Brennan et al. [56] reported that LX treatment decreased the development of proteinuria and glomerular injury in diabetic mice. It also repaired renal damage. In a separate study conducted by the same researchers, it was found that LXs could attenuate diabetic atherosclerotic lesions. Furthermore, their investigation revealed the mechanism underlying the action of LXs in vascular tissues. They discovered that LXs attenuate PDGF-stimulated vascular SMC proliferation, migration, and EC activation. Thus, LXs can be used as novel therapeutic agents to treat diabetes-related complications.

### 5.8. Neurological Disorders

Neurologic diseases involving the central and peripheral nervous systems are often associated with neuronal cell loss and axonal damage, which can cause permanent disability. LXs exhibit a range of protective effects in neurological conditions, including ischemic or hemorrhagic stroke, brain and spinal cord injury, Alzheimer’s disease, multiple sclerosis, and neuropathic pain. It is possible that some LXs or their derivatives may be used in the future as therapeutic agents for treating neurological diseases.

#### 5.8.1. Alzheimer’s Disease

Alzheimer’s disease (AD) is a neurological disorder that can lead to dementia. This condition was named after Alois Alzheimer, who first described it. Approximately 60–80% of dementia cases are classified as AD. It is a neurodegenerative disease characterized by symptoms of mood swings, agitation, memory loss, apathy, depression, delusion, hallucinations, and speech and visuospatial orientation. Two types of lesions are hallmarks of AD, namely senile plaque and intracellular neurofibrillary tangles (NFT). Deposits of β-amyloid peptides (Aβ) result in senile plaque, whereas hyperphosphorylation of the tau protein causes NFT [163]. Lower levels of LXA4 were found in the brains of 3xTg-AD mice compared with the control mice, suggesting that decreased LXA4 production may play a significant role in AD progression. It was demonstrated that AD-affected mouse brains exhibited increased markers of inflammation [164]. Notably, the same study demonstrated that AD mice treated with ATL exhibited reduced Aβ levels, decreased phosphorylation of tau, and enhanced cognitive performance. The authors provided evidence that decreased p-tau levels were associated with reduced activity of tau kinases GSK-3β and p38 MAPK [164].

It was also reported that LXA4 may play a neuroprotective role by allosterically enhancing the cannabinoid 1 receptor-dependent pathway and decreasing Aβ1–40 in the cortex and hippocampus of mice, decreasing AD pathology [165]. LXA4 was also shown to regulate redox-sensitive proteins, namely heat shock protein 72 and HO-1, thereby lessening neuroinflammation in rats [166]. Neurons from AD patients and APP/PS1 mice exhibited a decreased activity of neural sphingosine kinase 1, which is important for acetylating COX2 at serine residue 565. Consequently, lower concentrations of 15-R-LXA4 were found in the models. This was associated with defective microglial phagocytosis and inflammation resolution, leading to neuronal dysfunction [167]. Consistently, reduced LXA4 levels were observed in the CSF and hippocampus of AD-affected patients [168]. LXs and other SPMs significantly decrease neuroinflammation in AD. For example, combined treatment with LXA4 and resolvin E1 reduced the number of Aβ plaques and the Aβ-associated neuroinflammation compared with LXA4 alone [169].

#### 5.8.2. Multiple Sclerosis (MS)

MS is a chronic autoimmune neurological disorder that predominantly impacts the central nervous system. The primary cause of inflammation is focal T-lymphocytic and macrophage infiltration, which leads to the destruction of the myelin sheath and formation of MS plaques in the central nervous system. Symptoms include motor sensory issues, ataxia, and cognitive dysfunctions [170,171]. MS is more common in the 20–40 years age group and is more prevalent in females [172]. Lower SPM levels, including LXA4 and LXB4, were observed in patients with MS. Although LXA4 and LXB4 inhibited the transmigration of monocytes and decreased pro-inflammatory cytokine production in vitro [173], in vivo studies showed no benefits of LXA4 supplementation in an experimental autoimmune encephalomyelitis (EAE) mouse model of MS [174].

#### 5.8.3. Ischemic Stroke

Ischemic stroke is caused by the blockage of blood flow to the brain. This can be due to thrombotic or embolic events. Embolic events are caused by debris/clots from another location in the body that reach blood vessels in the brain, thereby blocking or slowing blood flow [175]. In contrast, thrombosis is facilitated by vascular wall inflammation. Stroke is one of the major causes of death and disability worldwide, with 68% of strokes being ischemic [176,177]. Ischemic strokes are classified into two types: large-vessel stroke and small-vessel stroke, or lacunar stroke [178]. LXA4 levels were elevated 1 h after middle cerebral artery occlusion (MCAO) in mice. An effect that lasted for 24 h [179]. A similarly elevated level was found in rats affected by global cerebral ischemia (GCI), which remained elevated for longer than 168 h [180]. Sobrado et al. demonstrated that LXA4 can activate anti-inflammatory nuclear receptor PPARγ and that LXA4 treatment (1 nmol) significantly reduced infarct volume and decreased neurological deficit scores following MCAO [181]. Sobrado et al. also showed that PPARγ agonist rosiglitazone increased 5-lipoxygenase expression and the production of LXA4 in ischemic rat brain, indicating that PPARγ can upregulate the release of its own agonist. LXA4 Methyl Ester (LXA4 ME) was shown to inhibit activation of microglia, neutrophil infiltration, lipid peroxidation, and it reduced the release of pro-inflammatory cytokines in a model of permanent cerebral ischemia by blocking the NF-κΒ pathway [182]. LXA4 ME also decreased the permeability of the blood–brain barrier after ischemic stroke by upregulating tissue metallopeptidase inhibitor-1 and downregulating matrix metallopeptidase 9 (MMP-9) [183]. LXA4 regulates microglial polarization by modulating the Notch signaling pathway [184]. Furthermore, it was demonstrated that LXA4 suppressed the phosphorylation of extracellular signal-regulated kinase (ERK) associated with middle cerebral artery occlusion and decreases the biosynthesis of leukotrienes, leading to neuroprotective and anti-inflammatory effects [185]. Pretreatment with LXA4 post-global cerebral ischemia–reperfusion showed improved mental abilities in aged rats [186]. Depression and loss of cognitive ability are common post-stroke issues in patients, and shifts in Beck Depression Inventory-II scores showed an inverse correlation with LXA4 and protectin D1 levels, indicating a possible beneficial role for LXA4 supplementation in treating post-stroke cognitive impairment and depression [187].

#### 5.8.4. Hemorrhagic Stroke

Hemorrhagic stroke is caused by the rupture of blood vessels in the brain. It is classified into two subtypes: intracerebral hemorrhage (ICH) and subarachnoid hemorrhage (SAH). The former involves bleeding into the brain parenchyma and later into the subarachnoid space, which is associated with high mortality rates [188]. Hypertension, cerebral amyloid angiopathy, cigarette smoking, and cerebral microbleeds are common risk factors [189,190]. LXA4 administration post-SAH in rats upregulated formyl peptide receptor 2 and inhibited the p38 MAPK signaling pathway, alleviating the deleterious consequences of SAH. Exogenous LXA4 also helped mitigate brain swelling, maintain the integrity of the blood–brain barrier, enhance neurological performance, and improve spatial learning and memory capabilities [191]. In a rat model of intracerebral hemorrhage (ICH), LXs inhibited neuronal apoptosis, lowered pro-inflammatory cytokine levels, and enhanced neurological function by suppressing the NF-kB-dependent MMP-9 pathway [192].

#### 5.8.5. Spinal Cord Injury (SCI)

The disruption or loss of motor and sensory function of spinal cord axons is a severe condition that leads to permanent disability in extreme cases [193]. Spinal cord injury can result from direct trauma to the spinal cord, compression arising from fractured vertebrae, or the presence of masses like epidural hematomas or abscesses. Rarely, factors such as restricted blood flow, inflammation, metabolic imbalance, and exposure to toxins may also lead to SCI [194]. LXA4 suppressed microglial activation, mitigated neuroinflammation, and reduced mechanical allodynia in spinal cord hemi-section [195]. Additionally, LXA4 was shown to decrease radicular pain following non-compressive lumbar disk herniation, possibly by inhibiting ERK, JNK, NF-κB/p65, and pro-inflammatory cytokine signaling in rats [196]. It was also shown that spinal delivery of LXA4 reduced inflammation-induced pain, likely through inhibition of ERK and JNK signaling in rat spinal astrocytes [197]. LXA4 also increased Akt/Nrf2/HO-1 signaling, enhanced functional recovery, alleviated allodynia and hyperalgesia, and led to lesion recovery and attenuation of apoptotic signaling [198].

#### 5.8.6. Neonatal Hypoxia–Ischemia Encephalopathy

Neonatal hypoxic–ischemic encephalopathy (HIE) may lead to cerebral palsy (CP) and other significant neurological impairments in children. This condition arises from insufficient blood flow and oxygen delivery to the brain, resulting in focal or diffuse brain damage. HIE in neonates is reported at a rate of 1.5 per 1000 live births [199]. LXA4 administration in a neonatal rat model of HI brain injury inhibited immediate inflammation and oxidative stress after brain injury and safeguarded against blood–brain barrier disruption by controlling the IκB/NF-κB signaling pathway. This, in turn, mitigated damage in the context of hypoxic–ischemic injury. In addition, LXA4 also showed promising results in achieving sustained neuroprotection by facilitating the restoration of neuronal function and tissue architecture observed seven days after post-hypoxic–ischemic injury (HII) and improving motor and learning functions 21 days post-HII [200].

#### 5.8.7. Traumatic Brain Injury

Traumatic brain injury (TBI) leads to significant neuroinflammation [201] and has diverse manifestations, ranging from mild alterations in consciousness to persistent coma or death. TBI can have lasting and evolving impacts, leading to persistent cognitive and psychiatric disorders, chronic headaches, nociceptive sensitization, disruptions in sleep–wake patterns, and neurodegeneration. Treatment includes cognitive therapy sessions daily and extensive surgical interventions like bilateral decompressive craniectomies in severe cases [202,203,204]. Following traumatic brain injury (TBI), LXA4 therapy has shown significant neuroprotective effects. Luo et al. provided evidence that the administration of LXA4 demonstrated an effective reduction in blood–brain barrier permeability and brain edema 24 h after TBI in mice. The neuroprotective effects of LXA4 are largely attributed to its inhibition of TNF-α, IL-1β, and IL-6 cytokine production and MAPK signaling, which are typically elevated post-injury [205]. Notably, Luo et al. also found that ALX/FPR2 was predominantly expressed on astrocytes rather than on microglia cells. Thus, these results suggest that astrocytes may be involved in LXA4-mediated neuroprotection [205].

Jung et al. evaluated whether LXA4 can be used as a biomarker of TBI [180]. Rats were subjected to TBI, and plasma levels of LXA4 and other specialized pro-resolving mediators (SPMs)—including RvE1, RvE2, RvD1, and RvD2—were measured at multiple time points (0, 6, 24, 72, and 168 h) post-injury. Jung et al. found a progressive decline in plasma LXA4 levels following TBI, contrasting with an increase observed in global cerebral ischemia (GCI) models, where LXA4 levels peaked at 24 and 72 h [180]. This decline in TBI animals indicated a failure to initiate effective neuroinflammation resolution. Meanwhile, other SPMs showed minimal changes, and IL-6 levels were significantly elevated, confirming a persistent pro-inflammatory state. These findings position LXA4 as a promising biomarker for TBI and underscore its potential diagnostic and therapeutic applications in acute traumatic brain injury [180].

#### 5.8.8. Neuropathic Pain

Aspirin-triggered lipoxin A4 (ATL) has demonstrated potent analgesic and anti-inflammatory properties. In a rat model of chronic constriction injury (CCI), intrathecal administration of ATL significantly alleviated mechanical allodynia and improved pain-related behaviors [206]. These effects were mediated through activation of the ALX/FPR2 receptor and were associated with a marked reduction in spinal levels of key pro-inflammatory cytokines, including IL-1β, IL-6, and TNF-α [206]. Furthermore, ATL inhibited the activation of the JAK2/STAT3 signaling pathway, which plays a central role in sustaining spinal neuroinflammation in neuropathic pain. Notably, the expression of SOCS3, a negative regulator of the JAK/STAT axis, was further enhanced when ATL was administered in combination with JAK2 and STAT3 inhibitors [206]. Collectively, these findings highlight ATL’s therapeutic potential in chronic pain management by suppressing inflammatory signaling and promoting anti-inflammatory feedback mechanisms [206].

**Figure 11:**
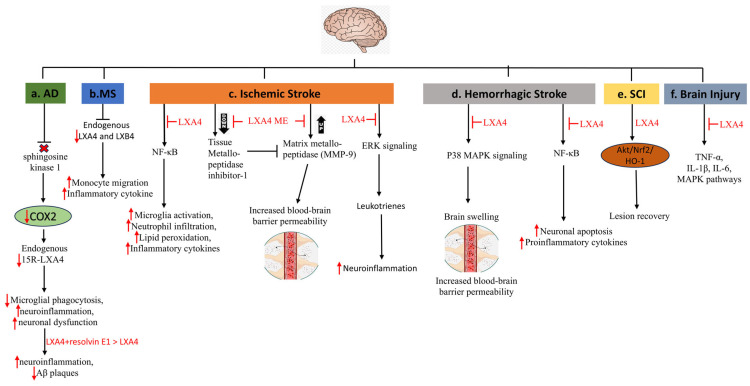
Lipoxins and neurological disorders. (**a**) In Alzheimer’s disease (AD), neural sphingosine kinase activity is low leading to reduced acetylation of COX2 and consequently impaired SPM secretion, leading to microglial phagocytosis and increased neuroinflammation. (**b**) Endogenous lipoxins levels are low in multiple sclerosis (MS), leading to increased monocyte migration and pro-inflammatory cytokine production. (**c**) After ischemic stroke, lipoxin-mediated blockage of the NF-κΒ signaling pathway leads to decreased microglia activation, neutrophil infiltration, cytokine release, and lipid peroxidation. Lipoxin-dependent upregulation of tissue metallopeptidase inhibitor-1 improves the integrity of the blood–brain barrier, whereas ERK pathway inhibition exhibits neuroprotective effects after ischemic stroke. (**d**) Downregulation of p38MAPK signaling pathway and inhibition of NF-kB by exogenous LXA4 decreased blood–brain barrier permeability and pro-inflammatory cytokine production in hemorrhagic stroke. (**e**) Upregulation of Akt/Nrf2/HO-1 signaling by LXA4 improves lesion recovery, hyperalgesia, and inhibition of apoptotic signaling in spinal cord injury (SCI); (**f**) LXA4 administration inhibits TNF-α, IL-1β, and IL-6 cytokine production after TBI. “↑” stands for upregulation; “↓” stands for downregulation; “Ⱶ” stands for inhibition.

Figure 11 summarizes the positive regulatory roles of lipoxin in various neurological disease pathways.

### 5.9. Cancer

The tumor microenvironment is crucial for cancer cell growth and migration. Tumor cells produce large amounts of vascular endothelial growth factor (VEGF) and TNF-α [207], which help them grow by promoting neoangiogenesis via the activation of vascular endothelial cells and by creating an inflammatory microenvironment [208]. LXs are known to affect the tumor immune microenvironment. For example, macrophages, neutrophils, and lymphocytes are crucial for cancer progression, and LXs can regulate these immune cells, influencing their functions during the body’s response to cancer [209,210].

The role of inflammation in cancer initiation and development was first realized by a German physician, Dr. Rudolf Virchow, in 1863. Later, it was estimated that up to 15% of cancer cases worldwide can be associated with either viral infection or chronic inflammation [211,212]. The unique characteristic of LXs, which distinguishes them from most other eicosanoid molecules, is their ability to reduce inflammation [15,213]. LXs were the first lipid mediators identified to play an essential role in the resolution phase of inflammation. LXA4 was found to (I) inhibit neutrophil chemotaxis, adherence, and transmigration; (II) attenuate the neutrophil activation process, including the inhibition of NF-kB activation reduction in superoxide generation and elastase secretion; (III) suppress IL-8 production by both epithelial cells and leukocytes; (IV) elevate bactericidal permeability-increasing protein (BPI) expression in epithelial cells; (V) upregulate chemotactic response in monocytes; (VI) upregulate monocyte ingestion of apoptotic neutrophils [15,213,214].

Insufficient production of natural anti-inflammatory compounds, such as LXs, that can resolve inflammation may potentially promote tumor formation and progression. Liu et al. provided evidence that LXA4 may have strong anti-cancer properties and could be a key molecule for managing inflammation-related cancers like colorectal cancer [66]. However, the authors also noted that LXA4 may have different roles during various tumor growth phases: exhibiting anti-cancer activity at the early stage and promoting tumor growth at late stages. The next section of this review will summarize the current knowledge about the LX roles in cancer pathophysiology.

#### 5.9.1. Pancreatic Cancer

Pancreatic cancer is the seventh leading cause of death worldwide and can be of adenocarcinomatous, serous, seromucous, or mucinous origin. Pancreatic cancer is difficult to diagnose at early stages because there are no significant symptoms until its very late stage. LXs are known to release pro-survival soluble molecules, reduce the synthesis and release of pro-inflammatory mediators, and inhibit inflammation, tumor cell proliferation, growth, and tumor invasiveness in part by the inhibition of autocrine TGF-β1 signaling [215] or by blocking the ROS/ERK/MMP pathway [216] in pancreatic cancers. It is also reported that LXs may have protective effects against experimental acute pancreatitis by inhibiting the production of pro-inflammatory cytokines [217]. Furthermore, LXs may have a direct antiangiogenic effect in pancreatic cancers by inhibiting vascular endothelial growth factor (VEGF) and hypoxia-inducible factor (HIF-1⍺) release, which are essential for tumor growth and progression [216].

#### 5.9.2. Kaposi’s Sarcoma (KS)

Kaposi sarcoma is a soft tissue tumor that usually develops from cells lining blood vessels and lymph nodes. It is locally progressive and has a high recurrence rate. It usually presents as a tumor on the skin, mucosal lining, and other body parts, such as the gastrointestinal tract, lymph nodes, and lungs. High levels of pro-inflammatory cytokines and proteins are a characteristic feature of Kaposi sarcoma. LX treatment decreased the expression of inflammatory proteins and cytokines and downregulated the critical signaling pathways involved in the disease [218]. LX-treated KS cells exhibited increased colocalization of ALXR with flotillin-1, suggesting an increased ALXR lipid raft localization and a decrease in lipid raft fractions. This may lead to decreased VEGFR-2 activity in KS cells. In the human Kaposi sarcoma tumor-derived cell line (KS-IMM), LXs also lowered the levels of pro-inflammatory prostaglandin E2 (PGE2) and LTB4. Additionally, lipoxin treatment decreased the secretion of the pro-inflammatory cytokines, such as IL-6 and IL-8, while inducing the secretion of the anti-inflammatory cytokine IL-10 [218]. Furthermore, LX treatment leads to reduced phosphorylation of VEGFR and ephrin family receptor tyrosine kinases, thereby inhibiting the angiogenic functions associated with Kaposi’s sarcoma [218].

#### 5.9.3. Colorectal Cancer

Colorectal cancer (CRC) is the third most common type of cancer and the fourth most common leading cause of cancer-related deaths on a global scale. Every year, more than a million new cases of CRC are detected globally [219]. Accumulated evidence from epidemiological, experimental, and clinical investigations has established a well-accepted association between inflammation and colorectal cancer, particularly in the context of IBD. It is estimated that infections and inflammatory reactions contribute to 15–20% of all cancer-related deaths worldwide [213]. It was reported that LXA4 deficiency may result in colorectal cancer development due to dysregulation in the tumor microenvironment [66]. Clinical samples and a mouse colorectal cell line (CT26) were used in this investigation. Thus, LXA4 is a potential target for modulating inflammation-associated cancers like colorectal cancer.

LXA4 inhibited the phosphorylation of p42/44 MAPK, Akt, and PLC-γ induced by VEGF [220,221]. Furthermore, it impeded the responsiveness of endothelial cells to VEGF, suppressing endothelial cell proliferation and neoangiogenesis [220,222,223]. LXA4 inhibited PDGF-induced cell proliferation and reduced PI3-kinase activity, PDGF-stimulated Akt/protein kinase B (PKB) activity, and TNF-α-activated NF-κB activity, all of which contributed to fibroblast apoptosis. LXA4 reduced the adhesion and activation of leukocytes induced by inflammatory mediators and neutrophil activation [214,217,224]. This action prevented acute tissue damage mediated by neutrophils. Furthermore, LXA4 modulated the PI3K/Akt and ERK/Nrf-2 defense pathways, thereby influencing apoptotic signaling in macrophages [7].

#### 5.9.4. Prostate Cancer

Prostate cancer is the second most common cancer type and one of the leading causes of death in men. Although prostate cancer is the slowest growing cancer in men, there are also aggressive types of prostate cancer that are accountable for about 15% of cases. Such aggressive prostate cancers may develop quickly. Prostate cancer cell survival and growth depend on testosterone derived from androgens. Therefore, androgen-deprivation therapy (or pharmacological castration) may be used to slow prostate cancer progression. It includes gonadotropin-releasing hormone agonists (leuprorelin), androgen biosynthesis inhibitors (abiraterone), and androgen receptor inhibitors (enzalutamide and apalutamide) [225]. However, prostate cancer eventually becomes insensitive to these inhibitors, transforming into the so-called castration-resistant prostate cancer. The role of LXs in prostate cancers is not fully understood. Tong and Tai reported that a combination treatment with IL-6 and androgens induced the nicotinamide adenine dinucleotide-dependent 15-hydroxyprostaglandin dehydrogenase (15-PGDH) expression in androgen-receptor positive LNCaP cells, a prostate cancer cell line [226]. 15-PGDH inactivates LXs, decreasing active LX levels in cultures. This effect might potentially promote the progression of androgen-sensitive prostate cancers. However, Tong and Tai did not investigate prostate cancer cell survival in their study. Also, an inverse relationship was reported between the expression level of 15-lipoxygenase-2, an enzyme critical for LX biosynthesis in prostate, and the stage of human prostate adenocarcinomas [227,228], suggesting that more advanced prostate cancers might have reduced tissue levels of LXs. On the other hand, Jia et al. demonstrated that LXA4 released from prostate cancer cells paradoxically promoted cancer cell survival, likely by inhibiting the RNA modification enzyme methyltransferase-like 3 (METTL3) via the STAT6 signaling pathway. The authors proposed that downregulation of METTL3 may result in a transformation of prostate cancer-associated macrophages from M1-like pro-inflammatory to M2-like anti-inflammatory type [229]. M2-like polarized cancer-associated macrophages are known to promote cancer cell survival. Indeed, in the same study, Jia et al. demonstrated that tumor size was reduced in NOD/SCID mice injected with human prostate adenocarcinoma LNCaP cells when the mice were treated four weeks with PBP10 (10 µg/mL), an ALX/FPR2 inhibitor, compared to the untreated LNCaP cell-injected NOD/SCID mice [229]. These data support the hypothesis that LXA4 can promote prostate cancer growth by activating the ALX/FPR2 receptor. Thus, paracrine release of LXA4 by prostate cancer cells may modulate the polarization of tumor-associated macrophages and promote tumorigenesis in prostate cancers.

#### 5.9.5. Breast Cancer

Breast cancer is one of the most common cancers among women, with the hormone receptor (estrogen and progesterone)-positive breast cancer being the most prevalent type. However, there are also hormone receptor-negative breast cancers, which are more difficult to treat. LXs appear to variably modulate the breast cancer microenvironment and thus influence cancer cell growth and migration.

Claria et al. reported that LXs inhibited A549 lung cancer cell proliferation in the following order 15-epi-LXB4 >>> LXB4 > 15-epi-LXA4 > LXA4 [230]. Later, Browne et al. provided evidence that LXA4 may suppress breast cancer cell migration using two breast cancer cell culture models, human MDA-MB-231 and mouse 4T1 breast cancer cell lines [231]. This effect was associated with a decreased expression of ICAM-1, CD11b, and matrix metalloproteinases (MMP-9) and increased expression of miRNA-146 [231]. Notably, miRNA-146 was already implicated in reducing breast cancer cell invasiveness in another in vitro model [232]. Consistently, Xu et al. reported that BML-111, a synthetic analog of LXA4, inhibited epithelial–mesenchymal transition and the migration of CoCl2-stimulated MCF-7 breast cancer cells by inhibiting MMP-2 and MMP-9, in part through downregulation of 5-lipoxygenase [233]. The authors found that BML-111 also inhibited the migration of MCF-7 breast cancer cells after inoculating them into BALB/c nude mice [233].

However, conversely, Khau et al. found that FPR2 agonists, such as LXA4, promoted proliferation of MCF-7 and MDA-MB-231 breast cancer cells via the pro-survival and pro-proliferation PI3K/Akt signaling pathway [234]. The authors found that FPR2 antagonists (WRW4 or Boc2) or siRNA-mediated downregulation of FPR2 decreased the proliferation rate of MCF-7 and MDA-MB-231 breast cancer cells [234]. A similar observation was made by Song et al., who demonstrated that the activation of the FPR2/ERK signaling pathway may promote breast cancer metastasis [235]. Thus, there is a complex interplay between anti-inflammatory and pro-survival actions of LXs. Likely, the effects depend on the involvement of specific subtypes of LXs and may be modulated by alterations in the LX receptor expression pattern in breast cancer cells. Notably, the latter can be epigenetically regulated [236].

#### 5.9.6. Aspirin as a Potential Cancer Therapy

Recent clinical evidence indicates that aspirin may reduce the risk of some cancer types. Rothwell et al. found that 75 mg daily of aspirin reduced the 20-year risk of incidence and mortality of proximal colon cancer but not distal colon cancer or rectal cancer in a cohort of 14,033 patients [237]. Consistent with these findings, Burn et al. later confirmed in a cohort of 861 patients with hereditary colorectal cancer (Lynch syndrome) that long-term treatment with 600 mg aspirin per day reduced cancer incidence [238,239]. Similar results were obtained by De Simoni et al., who demonstrated in the METACCRE cohort of 238 patients presenting with colorectal cancer that aspirin treatment was associated with a reduced number of nodal metastases and an increased number of tumor-infiltrating lymphocytes, suggesting that aspirin may enhance immunosurveillance against cancer [240]. Additionally, recent clinical studies and meta-analyses have revealed that long-term aspirin treatment can reduce prostate cancer incidence and associated mortality [241,242]. A meta-analysis of observational clinical studies, which were performed from 1989 to 2019 and enrolled 99,769 patients, also found that aspirin treatment reduced the risk of overall and in situ breast cancers [243]. However, more recent data indicate that daily aspirin did not improve the outcomes in high-risk nonmetastatic breast cancer patients [244]. Consistently, a meta-analysis of 67 randomized clinical trials also found no benefits of aspirin for the treatment or prevention of recurrence of breast, pancreatic, colorectal, lung, and prostate cancers [245]. Nevertheless, further research is needed to establish whether the aspirin-triggered LXs (15-epi-LXA4 or 15-epi-LXB4) contribute to the putative protective effect of aspirin at least against colorectal and prostate cancers. However, while considering aspirin-triggered LX effects in various cancer models, it is essential to differentiate them from other aspirin-dependent anti-cancer/anti-metastatic pathways. For example, it was reported that low-dose aspirin can reduce the frequency of lung metastases in a mouse model of B16 melanoma cell intravenous inoculations by releasing T cells from the thromboxane A2-dependent immunosuppression via the aspirin-dependent inhibition of COX-1-mediated thromboxane A2 production in platelets [246].

### 5.10. Endometriosis

Endometriosis is an inflammatory gynecological condition characterized by a significant increase in the hormone estrogen and resistance to progesterone [247,248]. Endometriosis can cause intense pain and infertility. It often goes undiagnosed until its severity has drastically increased. The only known treatments include invasive surgeries and hormone-based therapies that can have adverse future side effects. It was proposed that the upregulation of cyclooxygenase-2 (COX-2) may contribute, in part, to the development of endometriosis [249]. COX-2 drives prostaglandin E2 (PGE2) biosynthesis, and COX-2 expression can be induced by inflammation [250,251]. It has recently been observed that LXA4 can decrease the elevated levels of COX-2 in individuals presenting with endometriosis, likely by inhibiting p38 MAPK signaling [247]. p38 MAPK is a pro-inflammatory protein and tumor promoter, thus causing an inflammatory response, leading to endometriosis [252]. It was reported that treatment with 15-R-LXA4 reduced the severity of endometriosis in a mouse model [253], likely due to a decrease in IL-1β and TNF-α levels. These results suggest that 15-R-LXA4 may be a potential treatment for endometriosis [253]. Consistent results were obtained in a BALB/c mouse model of endometriosis [254]. The results revealed that LXA4 treatment decreased the inflammatory response and the phosphorylation of p38 MAPK.

### 5.11. Clinical Trails Related to LXs

Thus far, as of April 7, 2025, the ClinicalTrials.gov lists eight clinical studies related to LXs. One study investigated the effect of ticagrelor on 15-Epi-lipoxin A4 and inflammation during atherosclerosis (clinical study number: NCT02626169), which was withdrawn with no results published. Another clinical study investigated the effect of pioglitazone on the production of 15-EPI-lipoxin A4 in type 2 diabetes mellitus (clinical study number: NCT01040819). This small study, enrolling 25 subjects, was designed to determine whether pioglitazone can increase serum and urine levels of 15-epi-LXA4 in patients with diabetes mellitus type 2. Indeed, it was determined that treatment with 15 mg/day pioglitazone increased the urine 15-epi-LXA4 levels to 1 ng/mL.

The other clinical studies have addressed various oral diseases. A new clinical study, number NCT06789458, will assess the pro-resolution mediator levels in periodontitis stage III and IV before and after periodontal therapy. Four other oral disease-related clinical trials have been completed. These clinical studies investigated the effect of non-surgical periodontal treatment on lipoxin A4 levels (clinical trial number: NCT04053660), determined serum and saliva sirtuin 6, lipoxin A4, and caspase-8 levels in correlation with periodontal status in severe periodontitis (clinical trial number: NCT05417061), established lipoxin A4, annexin A1, and interleukin-1β levels in individuals with periodontitis (clinical trial number: NCT06700161), and evaluated the safety and preliminary efficacy of lipoxin analog BLXA4-ME oral rinse for the treatment of gingivitis (clinical trial number: NCT02342691).

Periodontitis is a chronic gum disease associated with bacterial- and host-mediated inflammation that may potentially lead to tooth loss. Three recently completed clinical studies have evaluated the levels of: LXA4, prostaglandin E2, and LTB4 (clinical trial number: NCT04053660); Sirtuin 6, LXA4, Caspase-8 (clinical trial number: NCT05417061); and Investigation of lipoxin A4, annexin A1, and IL-1β (clinical trial number: NCT06700161) in serum, gingival crevicular fluid, and saliva of individuals presenting with chronic periodontitis compared to healthy subjects. However, no results are available yet.

Clinical trial number NCT02342691 was a randomized, placebo-controlled, parallel-group phase 1 clinical trial. In the study, the 1 μM solution of BLXA4-ME [a LXA4 mimetic, methyl ester-benzo-lipoxin A4, or (5S, 6R, E)-methyl-5,6-dihydroxy-8-(2-((R,E)-3-hydroxyoct-1-enyl)phenyl)oct-7-enoate] was tested as a treatment for gingivitis. Hasturk et al. enrolled 123 subjects who were subdivided into three groups (one daily BLAX4-ME rinse, one daily vehicle rinse, and no rinse) and established that BLXA4 was safe and effective in reducing local gingival inflammation [255].

Clinical trial number NCT03609541 has been designed to assess the biomarkers during chronic obstructive pulmonary disease exacerbation. Specifically, the team focused on the serum amyloid A level and the serum amyloid A/LXA4 ratio. Amyloid A is a liver protein which is secreted during inflammatory diseases and can activate ALX/FPR2. Remarkably, amyloid A appears to be a biased agonist of ALX/FPR2, promoting the production of IL-8 and other pro-inflammatory mediators from airway epithelial cells in chronic obstructive pulmonary disease [256]. Conversely, LXA4, acting at the same ALX/FPR2 receptors, allosterically inhibits serum amyloid A-initiated pro-inflammatory responses, reducing the inflammatory response [256]. This study is still ongoing and recruits subjects. Therefore, no results have been published yet.

Thus, despite considerable advances in understanding the anti-inflammatory and pro-resolving roles of LXs and their analogs, only a few clinical trials attempt to translate the pre-clinical findings into the clinic. Therefore, there is an urgent need to do further clinical research to establish the clinical potential of LXs as therapeutic treatments.

## 6. Conclusions

Collectively, a growing body of evidence indicates that LXs are more than just anti-inflammatory agents. In this review, we have discussed the roles of LXs in various diseases and described their possible therapeutic effects. Although most of the published evidence supports the beneficial role of LXs in alleviating diseases, there are also reports indicating that LXs may worsen disease progression due to their pro-survival effect, specifically in the case of breast or prostate cancer.

There are some overlooked directions in the LX research field, such as the existence of paracrine effects of LXs released from prostate cancer cells on tumor-associated macrophage polarization, promoting cancer progression. Future pre-clinical studies are needed to obtain a deeper understanding of the precise molecular mechanisms across different disease contexts—especially in chronic inflammation, cancer, and autoimmune disorders. It is crucial to characterize the receptor–ligand dynamics, particularly involving ALX/FPR2, and identify potential co-receptors or modulators that influence LX signaling outcomes in different diseases.

On the therapeutic front, receptor-targeted therapies that selectively activate or modulate the ALX/FPR2 receptor present a promising approach, potentially offering greater specificity and reduced off-target effects. In addition, combination therapies—such as using lipoxins alongside corticosteroids, non-steroidal anti-inflammatory drugs (NSAIDs), or immunotherapies—may enhance beneficial outcomes. Advancing the design of stable lipoxin analogs with improved bioavailability, half-life, and targeted delivery systems (e.g., nanoparticles, liposomes) is also critical for clinical translation. These research avenues hold significant potential to position lipoxins as a next-generation platform for treating a range of inflammatory and immune-mediated diseases.

It is critical to further unravel the intricate signaling and regulatory events that govern LXs’ effects to utilize their full potential as therapeutic agents, while being cautious of their possible disease-promoting action. LXs may play a vital role as autacoids in controlling inflammation and orchestrating its resolution. However, further research is needed to better understand the contribution of changes in the differential expression of various LX receptors and to scrutinize the importance of specific subtypes of LXs in health and disease.

## Data Availability

All data and material are included within the review.

## Abbreviations

The following abbreviations are used in this manuscript:

AA	Arachidonic acid
Akt	Protein kinase B
ALXR	Lipoxin A4 receptor
ANX-A1	Annexin A1
ATL	Aspirin triggered lipoxin
BCL2	The B-cell lymphoma-2
BBB	Brain-blood barrier
BPD	Bronchopulmonary dysplasia
CF	Cystic fibrosis
CFTR	Cystic fibrosis transmembrane conductance regulator
COVID-19	Coronavirus disease 2019
FPR	Formyl peptide receptor
GC	Glucocorticoids
IL-1β	Interleukin 1b
IL-6	Interleukin 6
KS	Kaposi’s sarcoma
LOX	Lipoxygenase
LTs	leukotrienes (LTs)
LTA4	Leukotriene A4
LTB4	Leukotriene B4
LX	Lipoxin
LXA4	Lipoxin A4
LXB4	Lipoxin B4
MAPK	Mitogen-activated protein kinase
NF-κB	Nuclear factor kappa-light-chain-enhancer of activated B cells
PGE2	Prostaglandin E2
PI3K	Phosphoinositide 3-kinase
PKC	Protein kinase C
PGDH	Prostaglandin dehydrogenase
SARS	Severe acute respiratory syndrome
SPMs	Specialized pro-resolving mediators
TGF-β	Transforming growth factor beta
TLR	Toll-like receptor
TNF-α	Tumor necrosis factor alpha
VEGFR-2	Vascular endothelial growth factor receptor 2

## References

[1] Serhan C.N., Hamberg M., Samuelsson B. (1984). Trihydroxytetraenes: A novel series of compounds formed from arachidonic acid in human leukocytes. Biochem. Biophys. Res. Commun..

[2] Serhan C.N., Hamberg M., Samuelsson B. (1984). Lipoxins: Novel series of biologically active compounds formed from arachidonic acid in human leukocytes. Proc. Natl. Acad. Sci. USA.

[3] Romano M., Cianci E., Simiele F., Recchiuti A. (2015). Lipoxins and aspirin-triggered lipoxins in resolution of inflammation. Eur. J. Pharmacol..

[4] Sharma N.P., Dong L., Yuan C., Noon K.R., Smith W.L. (2010). Asymmetric acetylation of the cyclooxygenase-2 homodimer by aspirin and its effects on the oxygenation of arachidonic, eicosapentaenoic, and docosahexaenoic acids. Mol. Pharmacol..

[5] Mulugeta S., Suzuki T., Hernandez N.T., Griesser M., Boeglin W.E., Schneider C. (2010). Identification and absolute configuration of dihydroxy-arachidonic acids formed by oxygenation of 5S-HETE by native and aspirin-acetylated COX-2. J. Lipid Res..

[6] Chandrasekharan J.A., Sharma-Walia N. (2015). Lipoxins: Nature’s way to resolve inflammation. J. Inflamm. Res..

[7] Prieto P., Cuenca J., Traves P.G., Fernandez-Velasco M., Martin-Sanz P., Bosca L. (2010). Lipoxin A4 impairment of apoptotic signaling in macrophages: Implication of the PI3K/Akt and the ERK/Nrf-2 defense pathways. Cell Death Differ..

[8] Godson C., Mitchell S., Harvey K., Petasis N.A., Hogg N., Brady H.R. (2000). Cutting edge: Lipoxins rapidly stimulate nonphlogistic phagocytosis of apoptotic neutrophils by monocyte-derived macrophages. J. Immunol..

[9] Gaudin A., Tolar M., Peters O.A. (2018). Lipoxin A(4) Attenuates the Inflammatory Response in Stem Cells of the Apical Papilla via ALX/FPR2. Sci. Rep..

[10] Ramon S., Bancos S., Serhan C.N., Phipps R.P. (2014). Lipoxin A(4) modulates adaptive immunity by decreasing memory B-cell responses via an ALX/FPR2-dependent mechanism. Eur. J. Immunol..

[11] Fu T., Mohan M., Brennan E.P., Woodman O.L., Godson C., Kantharidis P., Ritchie R.H., Qin C.X. (2020). Therapeutic Potential of Lipoxin A(4) in Chronic Inflammation: Focus on Cardiometabolic Disease. ACS Pharmacol. Transl. Sci..

[12] Eltay E.G., Van Dyke T. (2023). Resolution of inflammation in oral diseases. Pharmacol. Ther..

[13] Ferreira I., Falcato F., Bandarra N., Rauter A.P. (2022). Resolvins, Protectins, and Maresins: DHA-Derived Specialized Pro-Resolving Mediators, Biosynthetic Pathways, Synthetic Approaches, and Their Role in Inflammation. Molecules.

[14] Fiore S., Serhan C.N. (1990). Formation of lipoxins and leukotrienes during receptor-mediated interactions of human platelets and recombinant human granulocyte/macrophage colony-stimulating factor-primed neutrophils. J. Exp. Med..

[15] Serhan C.N. (1997). Lipoxins and novel aspirin-triggered 15-epi-lipoxins (ATL): A jungle of cell-cell interactions or a therapeutic opportunity?. Prostaglandins.

[16] Claria J., Serhan C.N. (1995). Aspirin triggers previously undescribed bioactive eicosanoids by human endothelial cell-leukocyte interactions. Proc. Natl. Acad. Sci. USA.

[17] Folco G., Murphy R.C. (2006). Eicosanoid transcellular biosynthesis: From cell-cell interactions to in vivo tissue responses. Pharmacol. Rev..

[18] Serhan C.N., Maddox J.F., Petasis N.A., Akritopoulou-Zanze I., Papayianni A., Brady H.R., Colgan S.P., Madara J.L. (1995). Design of lipoxin A4 stable analogs that block transmigration and adhesion of human neutrophils. Biochemistry.

[19] Samuelsson B., Dahlen S.E., Lindgren J.A., Rouzer C.A., Serhan C.N. (1987). Leukotrienes and lipoxins: Structures, biosynthesis, and biological effects. Science.

[20] Serhan C.N., Levy B.D., Clish C.B., Gronert K., Chiang N. (2000). Lipoxins, aspirin-triggered 15-epi-lipoxin stable analogs and their receptors in anti-inflammation: A window for therapeutic opportunity. Advances in Eicosanoid Research.

[21] Edenius C., Heidvall K., Lindgren J.A. (1988). Novel transcellular interaction: Conversion of granulocyte-derived leukotriene A4 to cysteinyl-containing leukotrienes by human platelets. Eur. J. Biochem..

[22] Qin C.X., Norling L.V., Vecchio E.A., Brennan E.P., May L.T., Wootten D., Godson C., Perretti M., Ritchie R.H. (2022). Formylpeptide receptor 2: Nomenclature, structure, signalling and translational perspectives: IUPHAR review 35. Br. J. Pharmacol..

[23] Cooray S.N., Gobbetti T., Montero-Melendez T., McArthur S., Thompson D., Clark A.J., Flower R.J., Perretti M. (2013). Ligand-specific conformational change of the G-protein-coupled receptor ALX/FPR2 determines proresolving functional responses. Proc. Natl. Acad. Sci. USA.

[24] Tylek K., Trojan E., Regulska M., Lacivita E., Leopoldo M., Basta-Kaim A. (2021). Formyl peptide receptor 2, as an important target for ligands triggering the inflammatory response regulation: A link to brain pathology. Pharmacol. Rep..

[25] Schaldach C.M., Riby J., Bjeldanes L.F. (1999). Lipoxin A4: A new class of ligand for the Ah receptor. Biochemistry.

[26] Sanchez-Garcia S., Jaen R.I., Fernandez-Velasco M., Delgado C., Bosca L., Prieto P. (2023). Lipoxin-mediated signaling: ALX/FPR2 interaction and beyond. Pharmacol. Res..

[27] Serhan C.N., Chiang N., Van Dyke T.E. (2008). Resolving inflammation: Dual anti-inflammatory and pro-resolution lipid mediators. Nat. Rev. Immunol..

[28] Zhang J., Li Z., Fan M., Jin W. (2022). Lipoxins in the Nervous System: Brighter Prospects for Neuroprotection. Front. Pharmacol..

[29] Wu J., Ding D.H., Li Q.Q., Wang X.Y., Sun Y.Y., Li L.J. (2019). Lipoxin A4 Regulates Lipopolysaccharide-Induced BV2 Microglial Activation and Differentiation via the Notch Signaling Pathway. Front. Cell. Neurosci..

[30] Wang Y.P., Wu Y., Li L.Y., Zheng J., Liu R.G., Zhou J.P., Yuan S.Y., Shang Y., Yao S.L. (2011). Aspirin-triggered lipoxin A4 attenuates LPS-induced pro-inflammatory responses by inhibiting activation of NF-kappaB and MAPKs in BV-2 microglial cells. J. Neuroinflamm..

[31] Maderna P., Cottell D.C., Toivonen T., Dufton N., Dalli J., Perretti M., Godson C. (2010). FPR2/ALX receptor expression and internalization are critical for lipoxin A4 and annexin-derived peptide-stimulated phagocytosis. FASEB J..

[32] Cruz-Topete D., Cidlowski J.A. (2015). One hormone, two actions: Anti- and pro-inflammatory effects of glucocorticoids. Neuroimmunomodulation.

[33] Hashimoto A., Murakami Y., Kitasato H., Hayashi I., Endo H. (2007). Glucocorticoids co-interact with lipoxin A4 via lipoxin A4 receptor (ALX) up-regulation. Biomed. Pharmacother..

[34] Qin C., Yang Y.H., May L., Gao X., Stewart A.G., Tu Y., Woodman O.L., Ritchie R.H. (2015). Cardioprotective potential of annexin-A1 mimetics in myocardial infarction. Pharmacol. Ther..

[35] Serhan C.N., Levy B.D. (2018). Resolvins in inflammation: Emergence of the pro-resolving superfamily of mediators. J. Clin. Investig..

[36] Colby J.K., Gott K.M., Wilder J.A., Levy B.D. (2016). Lipoxin Signaling in Murine Lung Host Responses to Cryptococcus neoformans Infection. Am. J. Respir. Cell Mol. Biol..

[37] Kraft J.D., Blomgran R., Bergstrom I., Sotak M., Clark M., Rani A., Rajan M.R., Dalli J., Nystrom S., Quiding-Jarbrink M. (2022). Lipoxins modulate neutrophil oxidative burst, integrin expression and lymphatic transmigration differentially in human health and atherosclerosis. FASEB J..

[38] Petri M.H., Laguna-Fernandez A., Gonzalez-Diez M., Paulsson-Berne G., Hansson G.K., Back M. (2015). The role of the FPR2/ALX receptor in atherosclerosis development and plaque stability. Cardiovasc. Res..

[39] Peng C., Vecchio E.A., Nguyen A.T.N., De Seram M., Tang R., Keov P., Woodman O.L., Chen Y.C., Baell J., May L.T. (2024). Biased receptor signalling and intracellular trafficking profiles of structurally distinct formylpeptide receptor 2 agonists. Br. J. Pharmacol..

[40] Dufton N., Hannon R., Brancaleone V., Dalli J., Patel H.B., Gray M., D’Acquisto F., Buckingham J.C., Perretti M., Flower R.J. (2010). Anti-inflammatory role of the murine formyl-peptide receptor 2: Ligand-specific effects on leukocyte responses and experimental inflammation. J. Immunol..

[41] Chiang N., Serhan C.N. (2017). Structural elucidation and physiologic functions of specialized pro-resolving mediators and their receptors. Mol. Asp. Med..

[42] Sordi R., Menezes-de-Lima O., Horewicz V., Scheschowitsch K., Santos L.F., Assreuy J. (2013). Dual role of lipoxin A4 in pneumosepsis pathogenesis. Int. Immunopharmacol..

[43] Gilroy D.W. (2005). The role of aspirin-triggered lipoxins in the mechanism of action of aspirin. Prostaglandins Leukot. Essent. Fat. Acids.

[44] Koudelka A., Buchan G.J., Cechova V., O’Brien J.P., Stevenson E.R., Uvalle C.E., Liu H., Woodcock S.R., Mullett S.J., Zhang C. (2025). Lipoxin A(4) yields an electrophilic 15-oxo metabolite that mediates FPR2 receptor-independent anti-inflammatory signaling. J. Lipid Res..

[45] Fierro I.M., Colgan S.P., Bernasconi G., Petasis N.A., Clish C.B., Arita M., Serhan C.N. (2003). Lipoxin A4 and aspirin-triggered 15-epi-lipoxin A4 inhibit human neutrophil migration: Comparisons between synthetic 15 epimers in chemotaxis and transmigration with microvessel endothelial cells and epithelial cells. J. Immunol..

[46] Andrews D., Godson C. (2021). Lipoxins and synthetic lipoxin mimetics: Therapeutic potential in renal diseases. Biochim. Biophys. Acta Mol. Cell Biol. Lipids.

[47] de Gaetano M., Butler E., Gahan K., Zanetti A., Marai M., Chen J., Cacace A., Hams E., Maingot C., McLoughlin A. (2019). Asymmetric synthesis and biological evaluation of imidazole- and oxazole-containing synthetic lipoxin A(4) mimetics (sLXms). Eur. J. Med. Chem..

[48] Maddox J.F., Colgan S.P., Clish C.B., Petasis N.A., Fokin V.V., Serhan C.N. (1998). Lipoxin B4 regulates human monocyte/neutrophil adherence and motility: Design of stable lipoxin B4 analogs with increased biologic activity. FASEB J..

[49] Takano T., Fiore S., Maddox J.F., Brady H.R., Petasis N.A., Serhan C.N. (1997). Aspirin-triggered 15-epi-lipoxin A4 (LXA4) and LXA4 stable analogues are potent inhibitors of acute inflammation: Evidence for anti-inflammatory receptors. J. Exp. Med..

[50] Hachicha M., Pouliot M., Petasis N.A., Serhan C.N. (1999). Lipoxin (LX)A4 and aspirin-triggered 15-epi-LXA4 inhibit tumor necrosis factor 1alpha-initiated neutrophil responses and trafficking: Regulators of a cytokine-chemokine axis. J. Exp. Med..

[51] Kieran N.E., Doran P.P., Connolly S.B., Greenan M.C., Higgins D.F., Leonard M., Godson C., Taylor C.T., Henger A., Kretzler M. (2003). Modification of the transcriptomic response to renal ischemia/reperfusion injury by lipoxin analog. Kidney Int..

[52] Clish C.B., O’Brien J.A., Gronert K., Stahl G.L., Petasis N.A., Serhan C.N. (1999). Local and systemic delivery of a stable aspirin-triggered lipoxin prevents neutrophil recruitment in vivo. Proc. Natl. Acad. Sci. USA.

[53] Bannenberg G., Moussignac R.L., Gronert K., Devchand P.R., Schmidt B.A., Guilford W.J., Bauman J.G., Subramanyam B., Perez H.D., Parkinson J.F. (2004). Lipoxins and novel 15-epi-lipoxin analogs display potent anti-inflammatory actions after oral administration. Br. J. Pharmacol..

[54] Sun Y.P., Tjonahen E., Keledjian R., Zhu M., Yang R., Recchiuti A., Pillai P.S., Petasis N.A., Serhan C.N. (2009). Anti-inflammatory and pro-resolving properties of benzo-lipoxin A(4) analogs. Prostaglandins Leukot. Essent. Fat. Acids.

[55] Brennan E.P., Mohan M., McClelland A., Tikellis C., Ziemann M., Kaspi A., Gray S.P., Pickering R., Tan S.M., Ali-Shah S.T. (2018). Lipoxins Regulate the Early Growth Response-1 Network and Reverse Diabetic Kidney Disease. J. Am. Soc. Nephrol..

[56] Brennan E.P., Mohan M., McClelland A., de Gaetano M., Tikellis C., Marai M., Crean D., Dai A., Beuscart O., Derouiche S. (2018). Lipoxins Protect Against Inflammation in Diabetes-Associated Atherosclerosis. Diabetes.

[57] Levy B.D., Bonnans C., Silverman E.S., Palmer L.J., Marigowda G., Israel E., Severe Asthma Research Program N.H.L., Blood I. (2005). Diminished lipoxin biosynthesis in severe asthma. Am. J. Respir. Crit. Care Med..

[58] Wang S., Qian X., Shen C., Sun Q., Jing Y., Liu B., Zhang K., Li M., Wang J., Zhou H. (2023). The protective effects of lipoxin A4 on type 2 diabetes mellitus: A Chinese prospective cohort study. Front Endocrinol.

[59] Borgeson E., Johnson A.M., Lee Y.S., Till A., Syed G.H., Ali-Shah S.T., Guiry P.J., Dalli J., Colas R.A., Serhan C.N. (2015). Lipoxin A4 Attenuates Obesity-Induced Adipose Inflammation and Associated Liver and Kidney Disease. Cell Metab..

[60] Van Dyke T.E. (2017). Pro-resolving mediators in the regulation of periodontal disease. Mol. Aspects Med..

[61] Xu Z., Zhao F., Lin F., Xiang H., Wang N., Ye D., Huang Y. (2014). Preeclampsia is associated with a deficiency of lipoxin A4, an endogenous anti-inflammatory mediator. Fertil. Steril..

[62] Lin F., Zeng P., Xu Z., Ye D., Yu X., Wang N., Tang J., Zhou Y., Huang Y. (2012). Treatment of Lipoxin A(4) and its analogue on low-dose endotoxin induced preeclampsia in rat and possible mechanisms. Reprod. Toxicol..

[63] He J., Pham T.L., Kakazu A.H., Ponnath A., Do K.V., Bazan H.E.P. (2023). Lipoxin A4 (LXA4) Reduces Alkali-Induced Corneal Inflammation and Neovascularization and Upregulates a Repair Transcriptome. Biomolecules.

[64] Conte F.P., Menezes-de-Lima O., Verri W.A., Cunha F.Q., Penido C., Henriques M.G. (2010). Lipoxin A(4) attenuates zymosan-induced arthritis by modulating endothelin-1 and its effects. Br. J. Pharmacol..

[65] Saraiva-Santos T., Zaninelli T.H., Manchope M.F., Andrade K.C., Ferraz C.R., Bertozzi M.M., Artero N.A., Franciosi A., Badaro-Garcia S., Staurengo-Ferrari L. (2023). Therapeutic activity of lipoxin A(4) in TiO(2)-induced arthritis in mice: NF-kappaB and Nrf2 in synovial fluid leukocytes and neuronal TRPV1 mechanisms. Front. Immunol..

[66] Liu H., Zeng J., Huang W., Xu Q., Ye D., Sun R., Zhang D. (2019). Colorectal Cancer Is Associated with a Deficiency of Lipoxin A(4), an Endogenous Anti-inflammatory Mediator. J. Cancer.

[67] Riordan J.R., Rommens J.M., Kerem B., Alon N., Rozmahel R., Grzelczak Z., Zielenski J., Lok S., Plavsic N., Chou J.L. (1989). Identification of the cystic fibrosis gene: Cloning and characterization of complementary DNA. Science.

[68] Cantin A.M., Hartl D., Konstan M.W., Chmiel J.F. (2015). Inflammation in cystic fibrosis lung disease: Pathogenesis and therapy. J. Cyst. Fibros..

[69] Van Itallie C.M., Fanning A.S., Bridges A., Anderson J.M. (2009). ZO-1 stabilizes the tight junction solute barrier through coupling to the perijunctional cytoskeleton. Mol. Biol. Cell.

[70] Higgins G., Fustero Torre C., Tyrrell J., McNally P., Harvey B.J., Urbach V. (2016). Lipoxin A4 prevents tight junction disruption and delays the colonization of cystic fibrosis bronchial epithelial cells by Pseudomonas aeruginosa. Am. J. Physiol. Lung Cell. Mol. Physiol..

[71] Buchanan P.J., McNally P., Harvey B.J., Urbach V. (2013). Lipoxin A(4)-mediated KATP potassium channel activation results in cystic fibrosis airway epithelial repair. Am. J. Physiol. Lung Cell. Mol. Physiol..

[72] Higgins G., Buchanan P., Perriere M., Al-Alawi M., Costello R.W., Verriere V., McNally P., Harvey B.J., Urbach V. (2014). Activation of P2RY11 and ATP release by lipoxin A4 restores the airway surface liquid layer and epithelial repair in cystic fibrosis. Am. J. Respir. Cell Mol. Biol..

[73] El Kebir D., Filep J.G. (2010). Role of neutrophil apoptosis in the resolution of inflammation. Sci. World J..

[74] Bonnans C., Gras D., Chavis C., Mainprice B., Vachier I., Godard P., Chanez P. (2007). Synthesis and anti-inflammatory effect of lipoxins in human airway epithelial cells. Biomed. Pharmacother..

[75] Bonnans C., Mainprice B., Chanez P., Bousquet J., Urbach V. (2003). Lipoxin A4 stimulates a cytosolic Ca2+ increase in human bronchial epithelium. J. Biol. Chem..

[76] Verriere V., Higgins G., Al-Alawi M., Costello R.W., McNally P., Chiron R., Harvey B.J., Urbach V. (2012). Lipoxin A4 stimulates calcium-activated chloride currents and increases airway surface liquid height in normal and cystic fibrosis airway epithelia. PLoS ONE.

[77] Hodges R.R., Li D., Shatos M.A., Bair J.A., Lippestad M., Serhan C.N., Dartt D.A. (2017). Lipoxin A(4) activates ALX/FPR2 receptor to regulate conjunctival goblet cell secretion. Mucosal Immunol..

[78] Christie P.E., Spur B.W., Lee T.H. (1992). The effects of lipoxin A4 on airway responses in asthmatic subjects. Am. Rev. Respir. Dis..

[79] Tian C., Gao F., Li X., Li Z. (2020). Icariside II attenuates eosinophils-induced airway inflammation and remodeling via inactivation of NF-kappaB and STAT3 in an asthma mouse model. Exp. Mol. Pathol..

[80] Kazani S., Planaguma A., Ono E., Bonini M., Zahid M., Marigowda G., Wechsler M.E., Levy B.D., Israel E. (2013). Exhaled breath condensate eicosanoid levels associate with asthma and its severity. J. Allergy Clin. Immunol..

[81] Liu Y., Wei L., He C., Chen R., Meng L. (2021). Lipoxin A4 inhibits ovalbumin-induced airway inflammation and airway remodeling in a mouse model of asthma. Chem. Biol. Interact..

[82] Kumar V. (2020). Pulmonary Innate Immune Response Determines the Outcome of Inflammation During Pneumonia and Sepsis-Associated Acute Lung Injury. Front. Immunol..

[83] Yang A., Yang X., Wang J., Wang X., Wu H., Fan L., Li H., Li J. (2021). Effects of the Tight Junction Protein CLDN6 on Cell Migration and Invasion in High-Grade Meningioma. World Neurosurg..

[84] Padua T.A., Torres N.D., Candea A.L.P., Costa M.F.S., Silva J.D., Silva-Filho J.L., Costa F.T.M., Rocco P.R.M., Souza M.C., Henriques M.G. (2018). Therapeutic effect of Lipoxin A(4) in malaria-induced acute lung injury. J. Leukoc. Biol..

[85] O’Reilly M., Sozo F., Harding R. (2013). Impact of preterm birth and bronchopulmonary dysplasia on the developing lung: Long-term consequences for respiratory health. Clin. Exp. Pharmacol. Physiol..

[86] Ji Y.D., Luo Z.L., Chen C.X., Li B., Gong J., Wang Y.X., Chen L., Yao S.L., Shang Y. (2018). BML-111 suppresses TGF-beta1-induced lung fi broblast activation in vitro and decreases experimental pulmonary fibrosis in vivo. Int. J. Mol. Med..

[87] Kindermann A., Binder L., Baier J., Gundel B., Simm A., Haase R., Bartling B. (2019). Severe but not moderate hyperoxia of newborn mice causes an emphysematous lung phenotype in adulthood without persisting oxidative stress and inflammation. BMC Pulm. Med..

[88] Wu Q., Chong L., Shao Y., Chen S., Li C. (2019). Lipoxin A4 reduces hyperoxia-induced lung injury in neonatal rats through PINK1 signaling pathway. Int. Immunopharmacol..

[89] Adu-Agyeiwaah Y., Grant M.B., Obukhov A.G. (2020). The Potential Role of Osteopontin and Furin in Worsening Disease Outcomes in COVID-19 Patients with Pre-Existing Diabetes. Cells.

[90] Obukhov A.G., Stevens B.R., Prasad R., Li Calzi S., Boulton M.E., Raizada M.K., Oudit G.Y., Grant M.B. (2020). SARS-CoV-2 Infections and ACE2: Clinical Outcomes Linked With Increased Morbidity and Mortality in Individuals With Diabetes. Diabetes.

[91] Attallah N.G.M., El-Kadem A.H., Negm W.A., Elekhnawy E., El-Masry T.A., Elmongy E.I., Altwaijry N., Alanazi A.S., Al-Hamoud G.A., Ragab A.E. (2021). Promising Antiviral Activity of Agrimonia pilosa Phytochemicals against Severe Acute Respiratory Syndrome Coronavirus 2 Supported with In Vivo Mice Study. Pharmaceuticals.

[92] Das U.N. (2021). Bioactive Lipids in COVID-19-Further Evidence. Arch. Med. Res..

[93] Pal A., Gowdy K.M., Oestreich K.J., Beck M., Shaikh S.R. (2020). Obesity-Driven Deficiencies of Specialized Pro-resolving Mediators May Drive Adverse Outcomes During SARS-CoV-2 Infection. Front. Immunol..

[94] Shah V.K., Firmal P., Alam A., Ganguly D., Chattopadhyay S. (2020). Overview of Immune Response During SARS-CoV-2 Infection: Lessons From the Past. Front. Immunol..

[95] Rex D.A.B., Dagamajalu S., Kandasamy R.K., Raju R., Prasad T.S.K. (2021). SARS-CoV-2 signaling pathway map: A functional landscape of molecular mechanisms in COVID-19. J. Cell Commun. Signal..

[96] Cao Y., Zhou X., Yin Z., Yu X., Yang Q., Guo Q., Tian D., Xiong X., Xu G., Kuang X. (2018). The anti-inflammatory effect of BML-111 on COPD may be mediated by regulating NLRP3 inflammasome activation and ROS production. Prostaglandins Other Lipid Mediat..

[97] Lindstrom M., DeCleene N., Dorsey H., Fuster V., Johnson C.O., LeGrand K.E., Mensah G.A., Razo C., Stark B., Varieur Turco J. (2022). Global Burden of Cardiovascular Diseases and Risks Collaboration, 1990–2021. J. Am. Coll. Cardiol..

[98] Di Cesare M., Perel P., Taylor S., Kabudula C., Bixby H., Gaziano T.A., McGhie D.V., Mwangi J., Pervan B., Narula J. (2024). The Heart of the World. Glob. Heart.

[99] Serna M.F., Mosquera Escudero M., Garcia-Perdomo H.A. (2023). Lipoxins and their relationship with inflammation-associated diseases. A systematic review. Obes. Res. Clin. Pract..

[100] Solanki K., Bezsonov E., Orekhov A., Parihar S.P., Vaja S., White F.A., Obukhov A.G., Baig M.S. (2024). Effect of reactive oxygen, nitrogen, and sulfur species on signaling pathways in atherosclerosis. Vasc. Pharmacol..

[101] Caso V.M., Manzo V., Pecchillo Cimmino T., Conti V., Caso P., Esposito G., Russo V., Filippelli A., Ammendola R., Cattaneo F. (2021). Regulation of Inflammation and Oxidative Stress by Formyl Peptide Receptors in Cardiovascular Disease Progression. Life.

[102] Perretti M., Godson C. (2020). Formyl peptide receptor type 2 agonists to kick-start resolution pharmacology. Br. J. Pharmacol..

[103] Petri M.H., Laguna-Fernandez A., Arnardottir H., Wheelock C.E., Perretti M., Hansson G.K., Back M. (2017). Aspirin-triggered lipoxin A4 inhibits atherosclerosis progression in apolipoprotein E(-/-) mice. Br. J. Pharmacol..

[104] Hashem S., Dougha A., Tuffery P. (2025). Ligand-Induced Biased Activation of GPCRs: Recent Advances and New Directions from In Silico Approaches. Molecules.

[105] Mai J., Liu W., Fang Y., Zhang S., Qiu Q., Yang Y., Wang X., Huang T., Zhang H., Xie Y. (2018). The atheroprotective role of lipoxin A(4) prevents oxLDL-induced apoptotic signaling in macrophages via JNK pathway. Atherosclerosis.

[106] Xu F., Zhang J., Zhou X., Hao H. (2022). Lipoxin A(4) and its analog attenuate high fat diet-induced atherosclerosis via Keap1/Nrf2 pathway. Exp. Cell Res..

[107] O’Brien C.W., Juraschek S.P., Wee C.C. (2019). Prevalence of Aspirin Use for Primary Prevention of Cardiovascular Disease in the United States: Results From the 2017 National Health Interview Survey. Ann. Intern. Med..

[108] Yuan Z., Lu Y., Wei J., Wu J., Yang J., Cai Z. (2020). Abdominal Aortic Aneurysm: Roles of Inflammatory Cells. Front. Immunol..

[109] Petri M.H., Thul S., Andonova T., Lindquist-Liljeqvist M., Jin H., Skenteris N.T., Arnardottir H., Maegdefessel L., Caidahl K., Perretti M. (2018). Resolution of Inflammation Through the Lipoxin and ALX/FPR2 Receptor Pathway Protects Against Abdominal Aortic Aneurysms. JACC Basic. Transl. Sci..

[110] Jaen R.I., Fernandez-Velasco M., Terron V., Sanchez-Garcia S., Zaragoza C., Canales-Bueno N., Val-Blasco A., Vallejo-Cremades M.T., Bosca L., Prieto P. (2020). BML-111 treatment prevents cardiac apoptosis and oxidative stress in a mouse model of autoimmune myocarditis. FASEB J..

[111] Reina-Couto M., Carvalho J., Valente M.J., Vale L., Afonso J., Carvalho F., Bettencourt P., Sousa T., Albino-Teixeira A. (2014). Impaired resolution of inflammation in human chronic heart failure. Eur. J. Clin. Investig..

[112] Shi Y., Pan H., Zhang H.Z., Zhao X.Y., Jin J., Wang H.Y. (2017). Lipoxin A4 mitigates experimental autoimmune myocarditis by regulating inflammatory response, NF-kappaB and PI3K/Akt signaling pathway in mice. Eur. Rev. Med. Pharmacol. Sci..

[113] Thakur M., Tupe R.S. (2023). Lipoxin and glycation in SREBP signaling: Insight into diabetic cardiomyopathy and associated lipotoxicity. Prostaglandins Other Lipid Mediat..

[114] Chen R., Li J., Zhou J., Wang Y., Zhao X., Li N., Liu W., Liu C., Zhou P., Chen Y. (2023). Prognostic impacts of Lipoxin A4 in patients with acute myocardial infarction: A prospective cohort study. Pharmacol. Res..

[115] Kain V., Liu F., Kozlovskaya V., Ingle K.A., Bolisetty S., Agarwal A., Khedkar S., Prabhu S.D., Kharlampieva E., Halade G.V. (2017). Resolution Agonist 15-epi-Lipoxin A(4) Programs Early Activation of Resolving Phase in Post-Myocardial Infarction Healing. Sci. Rep..

[116] Kang G.J., Kim E.J., Lee C.H. (2020). Therapeutic Effects of Specialized Pro-Resolving Lipids Mediators on Cardiac Fibrosis via NRF2 Activation. Antioxidants.

[117] Wallace J.L., Zamuner S.R., McKnight W., Dicay M., Mencarelli A., del Soldato P., Fiorucci S. (2004). Aspirin, but not NO-releasing aspirin (NCX-4016), interacts with selective COX-2 inhibitors to aggravate gastric damage and inflammation. Am. J. Physiol. Gastrointest. Liver Physiol..

[118] Das U.N. (2005). Can COX-2 inhibitor-induced increase in cardiovascular disease risk be modified by essential fatty acids?. J. Assoc. Physicians India.

[119] Weisman S.M., Graham D.Y. (2002). Evaluation of the benefits and risks of low-dose aspirin in the secondary prevention of cardiovascular and cerebrovascular events. Arch. Intern. Med..

[120] Tulowiecka N., Kotlega D., Bohatyrewicz A., Szczuko M. (2021). Could Lipoxins Represent a New Standard in Ischemic Stroke Treatment?. Int. J. Mol. Sci..

[121] Xu X., Gao W., Li L., Hao J., Yang B., Wang T., Li L., Bai X., Li F., Ren H. (2021). Annexin A1 protects against cerebral ischemia-reperfusion injury by modulating microglia/macrophage polarization via FPR2/ALX-dependent AMPK-mTOR pathway. J. Neuroinflamm..

[122] Kennedy N., Chambers S.T., Nolan I., Gallagher K., Werno A., Browne M., Stamp L.K. (2015). Native Joint Septic Arthritis: Epidemiology, Clinical Features, and Microbiological Causes in a New Zealand Population. J. Rheumatol..

[123] Khan F.Y., Abu-Khattab M., Baagar K., Mohamed S.F., Elgendy I., Anand D., Malallah H., Sanjay D. (2013). Characteristics of patients with definite septic arthritis at Hamad General Hospital, Qatar: A hospital-based study from 2006 to 2011. Clin. Rheumatol..

[124] Boff D., Oliveira V.L.S., Queiroz Junior C.M., Galvao I., Batista N.V., Gouwy M., Menezes G.B., Cunha T.M., Verri Junior W.A., Proost P. (2020). Lipoxin A(4) impairs effective bacterial control and potentiates joint inflammation and damage caused by Staphylococcus aureus infection. FASEB J..

[125] Blaho V.A., Zhang Y., Hughes-Hanks J.M., Brown C.R. (2011). 5-Lipoxygenase-deficient mice infected with Borrelia burgdorferi develop persistent arthritis. J. Immunol..

[126] Zhang Y., Olson R.M., Brown C.R. (2017). Macrophage LTB(4) drives efficient phagocytosis of Borrelia burgdorferi via BLT1 or BLT2. J. Lipid Res..

[127] Takai D., Nagase T., Shimizu T. (2004). New therapeutic key for cystic fibrosis: A role for lipoxins. Nat. Immunol..

[128] Machado F.S., Aliberti J. (2008). Role of lipoxin in the modulation of immune response during infection. Int. Immunopharmacol..

[129] Machado F.S., Johndrow J.E., Esper L., Dias A., Bafica A., Serhan C.N., Aliberti J. (2006). Anti-inflammatory actions of lipoxin A4 and aspirin-triggered lipoxin are SOCS-2 dependent. Nat. Med..

[130] Aliberti J., Serhan C., Sher A. (2002). Parasite-induced lipoxin A4 is an endogenous regulator of IL-12 production and immunopathology in Toxoplasma gondii infection. J. Exp. Med..

[131] Shirey K.A., Pletneva L.M., Puche A.C., Keegan A.D., Prince G.A., Blanco J.C., Vogel S.N. (2010). Control of RSV-induced lung injury by alternatively activated macrophages is IL-4R alpha-, TLR4-, and IFN-beta-dependent. Mucosal Immunol..

[132] Genis P., Jett M., Bernton E.W., Boyle T., Gelbard H.A., Dzenko K., Keane R.W., Resnick L., Mizrachi Y., Volsky D.J. (1992). Cytokines and arachidonic metabolites produced during human immunodeficiency virus (HIV)-infected macrophage-astroglia interactions: Implications for the neuropathogenesis of HIV disease. J. Exp. Med..

[133] Molina-Berrios A., Campos-Estrada C., Henriquez N., Faundez M., Torres G., Castillo C., Escanilla S., Kemmerling U., Morello A., Lopez-Munoz R.A. (2013). Protective role of acetylsalicylic acid in experimental Trypanosoma cruzi infection: Evidence of a 15-epi-lipoxin A(4)-mediated effect. PLoS Negl. Trop. Dis..

[134] How K.Y., Song K.P., Chan K.G. (2016). Porphyromonas gingivalis: An Overview of Periodontopathic Pathogen below the Gum Line. Front. Microbiol..

[135] Serhan C.N., Jain A., Marleau S., Clish C., Kantarci A., Behbehani B., Colgan S.P., Stahl G.L., Merched A., Petasis N.A. (2003). Reduced inflammation and tissue damage in transgenic rabbits overexpressing 15-lipoxygenase and endogenous anti-inflammatory lipid mediators. J. Immunol..

[136] Parolini C. (2025). Sepsis and high-density lipoproteins: Pathophysiology and potential new therapeutic targets. Biochim. Biophys. Acta Mol. Basis Dis..

[137] Gounni A.S., Lamkhioued B., Ochiai K., Tanaka Y., Delaporte E., Capron A., Kinet J.P., Capron M. (1994). High-affinity IgE receptor on eosinophils is involved in defence against parasites. Nature.

[138] Ashour D.S. (2015). Toll-like receptor signaling in parasitic infections. Expert. Rev. Clin. Immunol..

[139] Wu B., Walker J., Spur B., Rodriguez A., Yin K. (2015). Effects of Lipoxin A4 on antimicrobial actions of neutrophils in sepsis. Prostaglandins Leukot. Essent. Fat. Acids.

[140] Bafica A., Scanga C.A., Serhan C., Machado F., White S., Sher A., Aliberti J. (2005). Host control of Mycobacterium tuberculosis is regulated by 5-lipoxygenase-dependent lipoxin production. J. Clin. Investig..

[141] Bomfim C.C.B., Fisher L., Amaral E.P., Mittereder L., McCann K., Correa A.A.S., Namasivayam S., Swamydas M., Moayeri M., Weiss J.M. (2022). Mycobacterium tuberculosis Induces Irg1 in Murine Macrophages by a Pathway Involving Both TLR-2 and STING/IFNAR Signaling and Requiring Bacterial Phagocytosis. Front. Cell. Infect. Microbiol..

[142] Ma R., Wang M., Shi P., Xie X., Duan D., Meng S., Yuan Q., Wu Y., Wang J. (2023). Effect of lipoxin A4 on the osteogenic differentiation of periodontal ligament stem cells under lipopolysaccharide-induced inflammatory conditions. Eur. J. Oral. Sci..

[143] Cianci E., Recchiuti A., Trubiani O., Diomede F., Marchisio M., Miscia S., Colas R.A., Dalli J., Serhan C.N., Romano M. (2016). Human Periodontal Stem Cells Release Specialized Proresolving Mediators and Carry Immunomodulatory and Prohealing Properties Regulated by Lipoxins. Stem Cells Transl. Med..

[144] Stenke L., Reizenstein P., Lindgren J.A. (1994). Leukotrienes and lipoxins--new potential performers in the regulation of human myelopoiesis. Leuk. Res..

[145] Stenke L., Mansour M., Edenius C., Reizenstein P., Lindgren J.A. (1991). Formation and proliferative effects of lipoxins in human bone marrow. Biochem. Biophys. Res. Commun..

[146] Lindgren J.A., Stenke L., Mansour M., Edenius C., Lauren L., Nasman-Glaser B., Ericsson I., Reizenstein P. (1993). Formation and effects of leukotrienes and lipoxins in human bone marrow. J. Lipid Mediat..

[147] Romano M., Patruno S., Pomilio A., Recchiuti A. (2019). Proresolving Lipid Mediators and Receptors in Stem Cell Biology: Concise Review. Stem Cells Transl. Med..

[148] Desplat V., Dupuis F., Trimoreau F., Dulery C., Praloran V., Denizot Y. (1998). Effects of lipoxygenase metabolites of arachidonic acid on the growth of human mononuclear marrow cells and marrow stromal cell cultures. Mediat. Inflamm..

[149] Serhan C.N., Chiang N. (2004). Novel endogenous small molecules as the checkpoint controllers in inflammation and resolution: Entree for resoleomics. Rheum. Dis. Clin. N. Am..

[150] Huang X., Liao J., Feng F., Chen S., Liao E., Li D., Dai X., Dong J., Shao Y. (2023). Combined Application of Exosomes and FPR2 Agonist LXA4 in Controlling Fetal Membrane Inflammation and Promoting Fetal Membrane Tissue Repair. Reprod. Sci..

[151] Tan J.L., Tan Y.Z., Muljadi R., Chan S.T., Lau S.N., Mockler J.C., Wallace E.M., Lim R. (2017). Amnion Epithelial Cells Promote Lung Repair via Lipoxin A(4). Stem Cells Transl. Med..

[152] Murphy S., Lim R., Dickinson H., Acharya R., Rosli S., Jenkin G., Wallace E. (2011). Human amnion epithelial cells prevent bleomycin-induced lung injury and preserve lung function. Cell Transplant..

[153] Kim H., Park S.H., Han S.Y., Lee Y.S., Cho J., Kim J.M. (2020). LXA(4)-FPR2 signaling regulates radiation-induced pulmonary fibrosis via crosstalk with TGF-beta/Smad signaling. Cell Death Dis..

[154] Bai Y., Wang J., He Z., Yang M., Li L., Jiang H. (2019). Mesenchymal Stem Cells Reverse Diabetic Nephropathy Disease via Lipoxin A4 by Targeting Transforming Growth Factor beta (TGF-beta)/smad Pathway and Pro-Inflammatory Cytokines. Med. Sci. Monit..

[155] Wada K., Arita M., Nakajima A., Katayama K., Kudo C., Kamisaki Y., Serhan C.N. (2006). Leukotriene B4 and lipoxin A4 are regulatory signals for neural stem cell proliferation and differentiation. FASEB J..

[156] Xu Z., Zhao S., Zhou T., Liao T., Huang X., Xiang H., Zhang Q., Huang Y., Lin F., Ye D. (2019). Lipoxin A4 interferes with embryo implantation via suppression of epithelial-mesenchymal transition. Am. J. Reprod. Immunol..

[157] Jaen R.I., Sanchez-Garcia S., Fernandez-Velasco M., Bosca L., Prieto P. (2021). Resolution-Based Therapies: The Potential of Lipoxins to Treat Human Diseases. Front. Immunol..

[158] Elias I., Ferre T., Vila L., Munoz S., Casellas A., Garcia M., Molas M., Agudo J., Roca C., Ruberte J. (2016). ALOX5AP Overexpression in Adipose Tissue Leads to LXA4 Production and Protection Against Diet-Induced Obesity and Insulin Resistance. Diabetes.

[159] Das U.N. (2013). Arachidonic acid and lipoxin A4 as possible endogenous anti-diabetic molecules. Prostaglandins Leukot. Essent. Fat. Acids.

[160] Spite M., Claria J., Serhan C.N. (2014). Resolvins, specialized proresolving lipid mediators, and their potential roles in metabolic diseases. Cell Metab..

[161] Bathina S., Das U.N. (2019). PUFAs, BDNF and lipoxin A4 inhibit chemical-induced cytotoxicity of RIN5F cells in vitro and streptozotocin-induced type 2 diabetes mellitus in vivo. Lipids Health Dis..

[162] Schwab J.M., Serhan C.N. (2006). Lipoxins and new lipid mediators in the resolution of inflammation. Curr. Opin. Pharmacol..

[163] Querfurth H.W., LaFerla F.M. (2010). Alzheimer’s disease. N. Engl. J. Med..

[164] Dunn H.C., Ager R.R., Baglietto-Vargas D., Cheng D., Kitazawa M., Cribbs D.H., Medeiros R. (2015). Restoration of lipoxin A4 signaling reduces Alzheimer’s disease-like pathology in the 3xTg-AD mouse model. J. Alzheimers Dis..

[165] Pamplona F.A., Ferreira J., Menezes de Lima O., Duarte F.S., Bento A.F., Forner S., Villarinho J.G., Bellocchio L., Wotjak C.T., Lerner R. (2012). Anti-inflammatory lipoxin A4 is an endogenous allosteric enhancer of CB1 cannabinoid receptor. Proc. Natl. Acad. Sci. USA.

[166] Trovato A., Siracusa R., Di Paola R., Scuto M., Fronte V., Koverech G., Luca M., Serra A., Toscano M.A., Petralia A. (2016). Redox modulation of cellular stress response and lipoxin A4 expression by Coriolus versicolor in rat brain: Relevance to Alzheimer’s disease pathogenesis. Neurotoxicology.

[167] Lee J.Y., Han S.H., Park M.H., Baek B., Song I.S., Choi M.K., Takuwa Y., Ryu H., Kim S.H., He X. (2018). Neuronal SphK1 acetylates COX2 and contributes to pathogenesis in a model of Alzheimer’s Disease. Nat. Commun..

[168] Wang X., Zhu M., Hjorth E., Cortes-Toro V., Eyjolfsdottir H., Graff C., Nennesmo I., Palmblad J., Eriksdotter M., Sambamurti K. (2015). Resolution of inflammation is altered in Alzheimer’s disease. Alzheimers Dement..

[169] Kantarci A., Aytan N., Palaska I., Stephens D., Crabtree L., Benincasa C., Jenkins B.G., Carreras I., Dedeoglu A. (2018). Combined administration of resolvin E1 and lipoxin A4 resolves inflammation in a murine model of Alzheimer’s disease. Exp. Neurol..

[170] Loma I., Heyman R. (2011). Multiple sclerosis: Pathogenesis and treatment. Curr. Neuropharmacol..

[171] Compston A., Coles A. (2008). Multiple sclerosis. Lancet.

[172] Gadoth N. (2003). Multiple sclerosis in children. Brain Dev..

[173] Kooij G., Troletti C.D., Leuti A., Norris P.C., Riley I., Albanese M., Ruggieri S., Libreros S., van der Pol S.M.A., van Het Hof B. (2020). Specialized pro-resolving lipid mediators are differentially altered in peripheral blood of patients with multiple sclerosis and attenuate monocyte and blood-brain barrier dysfunction. Haematologica.

[174] Derada Troletti C., Enzmann G., Chiurchiu V., Kamermans A., Tietz S.M., Norris P.C., Jahromi N.H., Leuti A., van der Pol S.M.A., Schouten M. (2021). Pro-resolving lipid mediator lipoxin A(4) attenuates neuro-inflammation by modulating T cell responses and modifies the spinal cord lipidome. Cell Rep..

[175] Ntaios G. (2020). Embolic Stroke of Undetermined Source: JACC Review Topic of the Week. J. Am. Coll. Cardiol..

[176] Benjamin E.J., Blaha M.J., Chiuve S.E., Cushman M., Das S.R., Deo R., de Ferranti S.D., Floyd J., Fornage M., Gillespie C. (2017). Heart Disease and Stroke Statistics-2017 Update: A Report From the American Heart Association. Circulation.

[177] Lozano R., Naghavi M., Foreman K., Lim S., Shibuya K., Aboyans V., Abraham J., Adair T., Aggarwal R., Ahn S.Y. (2012). Global and regional mortality from 235 causes of death for 20 age groups in 1990 and 2010: A systematic analysis for the Global Burden of Disease Study 2010. Lancet.

[178] Adams H.P., Davis P.H., Leira E.C., Chang K.C., Bendixen B.H., Clarke W.R., Woolson R.F., Hansen M.D. (1999). Baseline NIH Stroke Scale score strongly predicts outcome after stroke: A report of the Trial of Org 10172 in Acute Stroke Treatment (TOAST). Neurology.

[179] Marcheselli V.L., Hong S., Lukiw W.J., Tian X.H., Gronert K., Musto A., Hardy M., Gimenez J.M., Chiang N., Serhan C.N. (2003). Novel docosanoids inhibit brain ischemia-reperfusion-mediated leukocyte infiltration and pro-inflammatory gene expression. J. Biol. Chem..

[180] Jung J.S., Kho A.R., Lee S.H., Choi B.Y., Kang S.H., Koh J.Y., Suh S.W., Song D.K. (2020). Changes in plasma lipoxin A4, resolvins and CD59 levels after ischemic and traumatic brain injuries in rats. Korean J. Physiol. Pharmacol..

[181] Sobrado M., Pereira M.P., Ballesteros I., Hurtado O., Fernandez-Lopez D., Pradillo J.M., Caso J.R., Vivancos J., Nombela F., Serena J. (2009). Synthesis of lipoxin A4 by 5-lipoxygenase mediates PPARgamma-dependent, neuroprotective effects of rosiglitazone in experimental stroke. J. Neurosci..

[182] Wu Y., Ye X.H., Guo P.P., Xu S.P., Wang J., Yuan S.Y., Yao S.L., Shang Y. (2010). Neuroprotective effect of lipoxin A4 methyl ester in a rat model of permanent focal cerebral ischemia. J. Mol. Neurosci..

[183] Wu Y., Wang Y.P., Guo P., Ye X.H., Wang J., Yuan S.Y., Yao S.L., Shang Y. (2012). A lipoxin A4 analog ameliorates blood-brain barrier dysfunction and reduces MMP-9 expression in a rat model of focal cerebral ischemia-reperfusion injury. J. Mol. Neurosci..

[184] Li Q.Q., Ding D.H., Wang X.Y., Sun Y.Y., Wu J. (2021). Lipoxin A4 regulates microglial M1/M2 polarization after cerebral ischemia-reperfusion injury via the Notch signaling pathway. Exp. Neurol..

[185] Wu L., Miao S., Zou L.B., Wu P., Hao H., Tang K., Zeng P., Xiong J., Li H.H., Wu Q. (2012). Lipoxin A4 inhibits 5-lipoxygenase translocation and leukotrienes biosynthesis to exert a neuroprotective effect in cerebral ischemia/reperfusion injury. J. Mol. Neurosci..

[186] Wu H.S., Guo P.P., Jin Z., Li X.Y., Yang X., Ke J.J., Wang Y.L., Feng X.B. (2018). Effects of Lipoxin A4 Pretreatment on Cognitive Function of Aged Rats after Global Cerebral Ischemia Reperfusion. Curr. Med. Sci..

[187] Kotlega D., Zembron-Lacny A., Golab-Janowska M., Nowacki P., Szczuko M. (2020). The Association of Free Fatty Acids and Eicosanoids with the Severity of Depressive Symptoms in Stroke Patients. Int. J. Mol. Sci..

[188] Unnithan A.K.A., Das J.M., Mehta P. (2024). Hemorrhagic Stroke. StatPearls.

[189] Kitagawa K. (2022). Blood pressure management for secondary stroke prevention. Hypertens. Res..

[190] Castello J.P., Pasi M., Kubiszewski P., Abramson J.R., Charidimou A., Kourkoulis C., DiPucchio Z., Schwab K., Anderson C.D., Gurol M.E. (2022). Cerebral Small Vessel Disease and Depression Among Intracerebral Hemorrhage Survivors. Stroke.

[191] Guo Z., Hu Q., Xu L., Guo Z.N., Ou Y., He Y., Yin C., Sun X., Tang J., Zhang J.H. (2016). Lipoxin A4 Reduces Inflammation Through Formyl Peptide Receptor 2/p38 MAPK Signaling Pathway in Subarachnoid Hemorrhage Rats. Stroke.

[192] Song Y., Yang Y., Cui Y., Gao J., Wang K., Cui J. (2019). Lipoxin A4 Methyl Ester Reduces Early Brain Injury by Inhibition of the Nuclear Factor Kappa B (NF-kappaB)-Dependent Matrix Metallopeptidase 9 (MMP-9) Pathway in a Rat Model of Intracerebral Hemorrhage. Med. Sci. Monit..

[193] Eckert M.J., Martin M.J. (2017). Trauma: Spinal Cord Injury. Surg. Clin. N. Am..

[194] Alizadeh A., Dyck S.M., Karimi-Abdolrezaee S. (2019). Traumatic Spinal Cord Injury: An Overview of Pathophysiology, Models and Acute Injury Mechanisms. Front. Neurol..

[195] Martini A.C., Berta T., Forner S., Chen G., Bento A.F., Ji R.R., Rae G.A. (2016). Lipoxin A4 inhibits microglial activation and reduces neuroinflammation and neuropathic pain after spinal cord hemisection. J. Neuroinflamm..

[196] Miao G.S., Liu Z.H., Wei S.X., Luo J.G., Fu Z.J., Sun T. (2015). Lipoxin A4 attenuates radicular pain possibly by inhibiting spinal ERK, JNK and NF-kappaB/p65 and cytokine signals, but not p38, in a rat model of non-compressive lumbar disc herniation. Neuroscience.

[197] Svensson C.I., Zattoni M., Serhan C.N. (2007). Lipoxins and aspirin-triggered lipoxin inhibit inflammatory pain processing. J. Exp. Med..

[198] Lu T., Wu X., Wei N., Liu X., Zhou Y., Shang C., Duan Y., Dong Y. (2018). Lipoxin A4 protects against spinal cord injury via regulating Akt/nuclear factor (erythroid-derived 2)-like 2/heme oxygenase-1 signaling. Biomed. Pharmacother..

[199] Kurinczuk J.J., White-Koning M., Badawi N. (2010). Epidemiology of neonatal encephalopathy and hypoxic-ischaemic encephalopathy. Early Hum. Dev..

[200] Zhu J.J., Yu B.Y., Fu C.C., He M.Z., Zhu J.H., Chen B.W., Zheng Y.H., Chen S.Q., Fu X.Q., Li P.J. (2020). LXA4 protects against hypoxic-ischemic damage in neonatal rats by reducing the inflammatory response via the IkappaB/NF-kappaB pathway. Int. Immunopharmacol..

[201] Nguyen T., Nguyen N., Cochran A.G., Smith J.A., Al-Juboori M., Brumett A., Saxena S., Talley S., Campbell E.M., Obukhov A.G. (2023). Repeated closed-head mild traumatic brain injury-induced inflammation is associated with nociceptive sensitization. J. Neuroinflamm..

[202] Galgano M., Toshkezi G., Qiu X., Russell T., Chin L., Zhao L.R. (2017). Traumatic Brain Injury: Current Treatment Strategies and Future Endeavors. Cell Transplant..

[203] Moretti L., Cristofori I., Weaver S.M., Chau A., Portelli J.N., Grafman J. (2012). Cognitive decline in older adults with a history of traumatic brain injury. Lancet Neurol..

[204] Sandsmark D.K., Elliott J.E., Lim M.M. (2017). Sleep-Wake Disturbances After Traumatic Brain Injury: Synthesis of Human and Animal Studies. Sleep.

[205] Luo C.L., Li Q.Q., Chen X.P., Zhang X.M., Li L.L., Li B.X., Zhao Z.Q., Tao L.Y. (2013). Lipoxin A4 attenuates brain damage and downregulates the production of pro-inflammatory cytokines and phosphorylated mitogen-activated protein kinases in a mouse model of traumatic brain injury. Brain Res..

[206] Wang Z.F., Li Q., Liu S.B., Mi W.L., Hu S., Zhao J., Tian Y., Mao-Ying Q.L., Jiang J.W., Ma H.J. (2014). Aspirin-triggered Lipoxin A4 attenuates mechanical allodynia in association with inhibiting spinal JAK2/STAT3 signaling in neuropathic pain in rats. Neuroscience.

[207] Terracciano A., Iacono D., O’Brien R.J., Troncoso J.C., An Y., Sutin A.R., Ferrucci L., Zonderman A.B., Resnick S.M. (2013). Personality and resilience to Alzheimer’s disease neuropathology: A prospective autopsy study. Neurobiol. Aging.

[208] Mantovani A., Allavena P., Sica A., Balkwill F. (2008). Cancer-related inflammation. Nature.

[209] Bray F., Ferlay J., Soerjomataram I., Siegel R.L., Torre L.A., Jemal A. (2018). Global cancer statistics 2018: GLOBOCAN estimates of incidence and mortality worldwide for 36 cancers in 185 countries. CA Cancer J. Clin..

[210] Zhang T., Hao H., Zhou X.Y. (2019). The role of lipoxin in regulating tumor immune microenvironments. Prostaglandins Other Lipid Mediat..

[211] Arbyn M., Weiderpass E., Bruni L., de Sanjose S., Saraiya M., Ferlay J., Bray F. (2020). Estimates of incidence and mortality of cervical cancer in 2018: A worldwide analysis. Lancet Glob. Health.

[212] Elinav E., Nowarski R., Thaiss C.A., Hu B., Jin C., Flavell R.A. (2013). Inflammation-induced cancer: Crosstalk between tumours, immune cells and microorganisms. Nat. Rev. Cancer.

[213] Kinlen L.J., Balkwill A. (2001). Infective cause of childhood leukaemia and wartime population mixing in Orkney and Shetland, UK. Lancet.

[214] Serhan C.N., Chiang N. (2008). Endogenous pro-resolving and anti-inflammatory lipid mediators: A new pharmacologic genus. Br. J. Pharmacol..

[215] Zong L., Chen K., Jiang Z., Chen X., Sun L., Ma J., Zhou C., Xu Q., Duan W., Han L. (2017). Lipoxin A4 reverses mesenchymal phenotypes to attenuate invasion and metastasis via the inhibition of autocrine TGF-beta1 signaling in pancreatic cancer. J. Exp. Clin. Cancer Res..

[216] Zong L., Li J., Chen X., Chen K., Li W., Li X., Zhang L., Duan W., Lei J., Xu Q. (2016). Lipoxin A4 Attenuates Cell Invasion by Inhibiting ROS/ERK/MMP Pathway in Pancreatic Cancer. Oxid. Med. Cell Longev..

[217] Varani J., Ward P.A. (1994). Mechanisms of endothelial cell injury in acute inflammation. Shock.

[218] Marginean A., Sharma-Walia N. (2015). Lipoxins exert antiangiogenic and anti-inflammatory effects on Kaposi’s sarcoma cells. Transl. Res..

[219] Morgan E., Arnold M., Gini A., Lorenzoni V., Cabasag C.J., Laversanne M., Vignat J., Ferlay J., Murphy N., Bray F. (2023). Global burden of colorectal cancer in 2020 and 2040: Incidence and mortality estimates from GLOBOCAN. Gut.

[220] Baker N., O’Meara S.J., Scannell M., Maderna P., Godson C. (2009). Lipoxin A4: Anti-inflammatory and anti-angiogenic impact on endothelial cells. J. Immunol..

[221] Fujio Y., Walsh K. (1999). Akt mediates cytoprotection of endothelial cells by vascular endothelial growth factor in an anchorage-dependent manner. J. Biol. Chem..

[222] Tsopanoglou N.E., Pipili-Synetos E., Maragoudakis M.E. (1994). Leukotrienes C4 and D4 promote angiogenesis via a receptor-mediated interaction. Eur. J. Pharmacol..

[223] Cezar-de-Mello P.F., Vieira A.M., Nascimento-Silva V., Villela C.G., Barja-Fidalgo C., Fierro I.M. (2008). ATL-1, an analogue of aspirin-triggered lipoxin A4, is a potent inhibitor of several steps in angiogenesis induced by vascular endothelial growth factor. Br. J. Pharmacol..

[224] Gil-Villa A.M., Norling L.V., Serhan C.N., Cordero D., Rojas M., Cadavid A. (2012). Aspirin triggered-lipoxin A4 reduces the adhesion of human polymorphonuclear neutrophils to endothelial cells initiated by preeclamptic plasma. Prostaglandins Leukot. Essent. Fat. Acids.

[225] Orme J.J., Pagliaro L.C., Quevedo J.F., Park S.S., Costello B.A. (2022). Rational Second-Generation Antiandrogen Use in Prostate Cancer. Oncologist.

[226] Tong M., Tai H.H. (2004). Synergistic induction of the nicotinamide adenine dinucleotide-linked 15-hydroxyprostaglandin dehydrogenase by an androgen and interleukin-6 or forskolin in human prostate cancer cells. Endocrinology.

[227] Shappell S.B., Boeglin W.E., Olson S.J., Kasper S., Brash A.R. (1999). 15-lipoxygenase-2 (15-LOX-2) is expressed in benign prostatic epithelium and reduced in prostate adenocarcinoma. Am. J. Pathol..

[228] Jack G.S., Brash A.R., Olson S.J., Manning S., Coffey C.S., Smith J.A., Shappell S.B. (2000). Reduced 15-lipoxygenase-2 immunostaining in prostate adenocarcinoma: Correlation with grade and expression in high-grade prostatic intraepithelial neoplasia. Hum. Pathol..

[229] Jia G., Wang X., Wu W., Zhang Y., Chen S., Zhao J., Zhao W., Li W., Sun X., Han B. (2022). LXA4 enhances prostate cancer progression by facilitating M2 macrophage polarization via inhibition of METTL3. Int. Immunopharmacol..

[230] Claria J., Lee M.H., Serhan C.N. (1996). Aspirin-triggered lipoxins (15-epi-LX) are generated by the human lung adenocarcinoma cell line (A549)-neutrophil interactions and are potent inhibitors of cell proliferation. Mol. Med..

[231] Browne E., Chen H., Vencken S., Condron C.M. (2020). Lipoxin A4 a novel therapeutic agent for the treatment of breast cancer metastases. Integr. Cancer Sci. Ther..

[232] Hurst D.R., Edmonds M.D., Scott G.K., Benz C.C., Vaidya K.S., Welch D.R. (2009). Breast cancer metastasis suppressor 1 up-regulates miR-146, which suppresses breast cancer metastasis. Cancer Res..

[233] Xu F., Zhou X., Lin L., Xu J., Feng Y., He Y., Hao H. (2023). BML-111, the agonist of lipoxin A4, suppresses epithelial-mesenchymal transition and migration of MCF-7 cells via regulating the lipoxygenase pathway. Int. J. Immunopathol. Pharmacol..

[234] Khau T., Langenbach S.Y., Schuliga M., Harris T., Johnstone C.N., Anderson R.L., Stewart A.G. (2011). Annexin-1 signals mitogen-stimulated breast tumor cell proliferation by activation of the formyl peptide receptors (FPRs) 1 and 2. FASEB J..

[235] Song L., Li H., Ma R.R., Liu S., Zhang G.H., Guo X.Y., Zhao R.N., Wu X.J., Zhang K., Gao P. (2022). E2F1-initiated transcription of PRSS22 promotes breast cancer metastasis by cleaving ANXA1 and activating FPR2/ERK signaling pathway. Cell Death Dis..

[236] Simiele F., Recchiuti A., Patruno S., Plebani R., Pierdomenico A.M., Codagnone M., Romano M. (2016). Epigenetic regulation of the formyl peptide receptor 2 gene. Biochim. Biophys. Acta.

[237] Rothwell P.M., Wilson M., Elwin C.E., Norrving B., Algra A., Warlow C.P., Meade T.W. (2010). Long-term effect of aspirin on colorectal cancer incidence and mortality: 20-year follow-up of five randomised trials. Lancet.

[238] Burn J., Gerdes A.M., Macrae F., Mecklin J.P., Moeslein G., Olschwang S., Eccles D., Evans D.G., Maher E.R., Bertario L. (2011). Long-term effect of aspirin on cancer risk in carriers of hereditary colorectal cancer: An analysis from the CAPP2 randomised controlled trial. Lancet.

[239] Burn J., Sheth H., Elliott F., Reed L., Macrae F., Mecklin J.P., Moslein G., McRonald F.E., Bertario L., Evans D.G. (2020). Cancer prevention with aspirin in hereditary colorectal cancer (Lynch syndrome), 10-year follow-up and registry-based 20-year data in the CAPP2 study: A double-blind, randomised, placebo-controlled trial. Lancet.

[240] Simoni O., Scarpa M., Castagliuolo I., Stepanyan A., Angriman I., Kotsafti A., Nacci C., Scognamiglio F., Negro S., D’Angelo A. (2024). IMMUNOREACT 7: Regular aspirin use is associated with immune surveillance activation in colorectal cancer. Cancer.

[241] Ma S., Xia W., Wu B., Sun C., Jiang Y., Liu H., Lowe S., Zhou Z., Xie P., Gao J. (2023). Effect of aspirin on incidence, recurrence, and mortality in prostate cancer patients: Integrating evidence from randomized controlled trials and real-world studies. Eur. J. Clin. Pharmacol..

[242] Koo H.Y., Jeong S.M., Cho M.H., Chun S., Shin D.W., Park J. (2021). Population-wide impacts of aspirin, statins, and metformin use on prostate cancer incidence and mortality. Sci. Rep..

[243] Ma S., Guo C., Sun C., Han T., Zhang H., Qu G., Jiang Y., Zhou Q., Sun Y. (2021). Aspirin Use and Risk of Breast Cancer: A Meta-analysis of Observational Studies from 1989 to 2019. Clin. Breast Cancer.

[244] Chen W.Y., Ballman K.V., Partridge A.H., Hahn O.M., Briccetti F.M., Irvin W.J., Symington B., Visvanathan K., Pohlmann P.R., Openshaw T.H. (2024). Aspirin vs Placebo as Adjuvant Therapy for Breast Cancer: The Alliance A011502 Randomized Trial. JAMA.

[245] Benjamin D.J., Haslam A., Prasad V. (2024). Cardiovascular/anti-inflammatory drugs repurposed for treating or preventing cancer: A systematic review and meta-analysis of randomized trials. Cancer Med..

[246] Yang J., Yamashita-Kanemaru Y., Morris B.I., Contursi A., Trajkovski D., Xu J., Patrascan I., Benson J., Evans A.C., Conti A.G. (2025). Aspirin prevents metastasis by limiting platelet TXA(2) suppression of T cell immunity. Nature.

[247] Dai S., Zhu M., Wu R., Lin D., Huang Z., Ren L., Huang S., Cheng L., Chen Q. (2019). Lipoxin A(4) Suppresses IL-1beta-Induced Cyclooxygenase-2 Expression Through Inhibition of p38 MAPK Activation in Endometriosis. Reprod. Sci..

[248] Young S.L., Lessey B.A. (2010). Progesterone function in human endometrium: Clinical perspectives. Semin. Reprod. Med..

[249] Lai Z.Z., Yang H.L., Ha S.Y., Chang K.K., Mei J., Zhou W.J., Qiu X.M., Wang X.Q., Zhu R., Li D.J. (2019). Cyclooxygenase-2 in Endometriosis. Int. J. Biol. Sci..

[250] Takenaka Y., Taniguchi F., Miyakoda H., Takai E., Terakawa N., Harada T. (2010). Lipopolysaccharide promoted proliferation and invasion of endometriotic stromal cells via induction of cyclooxygenase-2 expression. Fertil. Steril..

[251] Binion D.G., Otterson M.F., Rafiee P. (2008). Curcumin inhibits VEGF-mediated angiogenesis in human intestinal microvascular endothelial cells through COX-2 and MAPK inhibition. Gut.

[252] Martinez-Limon A., Joaquin M., Caballero M., Posas F., de Nadal E. (2020). The p38 Pathway: From Biology to Cancer Therapy. Int. J. Mol. Sci..

[253] Chen Q., Zhou W., Pu D., Li Z., Huang Q., Chen Q. (2009). The inhibitory effect of 15-R-LXA4 on experimental endometriosis. Eur. J. Obstet. Gynecol. Reprod. Biol..

[254] Wu R., Zhou W., Chen S., Shi Y., Su L., Zhu M., Chen Q., Chen Q. (2014). Lipoxin A4 suppresses the development of endometriosis in an ALX receptor-dependent manner via the p38 MAPK pathway. Br. J. Pharmacol..

[255] Hasturk H., Schulte F., Martins M., Sherzai H., Floros C., Cugini M., Chiu C.J., Hardt M., Van Dyke T. (2021). Safety and Preliminary Efficacy of a Novel Host-Modulatory Therapy for Reducing Gingival Inflammation. Front. Immunol..

[256] Bozinovski S., Uddin M., Vlahos R., Thompson M., McQualter J.L., Merritt A.S., Wark P.A., Hutchinson A., Irving L.B., Levy B.D. (2012). Serum amyloid A opposes lipoxin A(4) to mediate glucocorticoid refractory lung inflammation in chronic obstructive pulmonary disease. Proc. Natl. Acad. Sci. USA.

